# Beyond HRV: attractor reconstruction using the entire cardiovascular waveform data for novel feature extraction

**DOI:** 10.1088/1361-6579/aaa93d

**Published:** 2018-03-01

**Authors:** Philip J Aston, Mark I Christie, Ying H Huang, Manasi Nandi

**Affiliations:** 1 Department of Mathematics, University of Surrey, Guildford, Surrey GU2 7XH, United Kingdom; 2 Institute of Pharmaceutical Science, Faculty of Life Sciences and Medicine, King’s College London, Franklin Wilkins Building, 150 Stamford Street, London SE1 9NH, United Kingdom; 3 Cardiovascular Division, Faculty of Life Sciences and Medicine, King’s College London, Franklin Wilkins Building, 150 Stamford Street, London SE1 9NH, United Kingdom; P.Aston@surrey.ac.uk

**Keywords:** blood pressure data, attractor reconstruction, baseline variation, feature extraction, heart rate variability

## Abstract

Advances in monitoring technology allow blood pressure waveforms to be collected at sampling frequencies of 250–1000 Hz for long time periods. However, much of the raw data are under-analysed. Heart rate variability (HRV) methods, in which beat-to-beat interval lengths are extracted and analysed, have been extensively studied. However, this approach discards the majority of the raw data. *Objective*: Our aim is to detect changes in the shape of the waveform in long streams of blood pressure data. *Approach*: Our approach involves extracting key features from large complex data sets by generating a reconstructed attractor in a three-dimensional phase space using delay coordinates from a window of the entire raw waveform data. The naturally occurring baseline variation is removed by projecting the attractor onto a plane from which new quantitative measures are obtained. The time window is moved through the data to give a collection of signals which relate to various aspects of the waveform shape. *Main results*: This approach enables visualisation and quantification of changes in the waveform shape and has been applied to blood pressure data collected from conscious unrestrained mice and to human blood pressure data. The interpretation of the attractor measures is aided by the analysis of simple artificial waveforms. *Significance*: We have developed and analysed a new method for analysing blood pressure data that uses all of the waveform data and hence can detect changes in the waveform shape that HRV methods cannot, which is confirmed with an example, and hence our method goes ‘beyond HRV’.

## 1. Introduction

The cardiovascular system keeps blood in continuous motion around the body ensuring that adequate cellular oxygen and nutrient requirements are met at any given time. The system must be able to adapt to acute changes in the body’s physiology such as sleep, postural changes and exercise (Dampney *et al* 2002). Analysis of the blood pressure (BP) signal, which is an approximately periodic waveform, allows quantification of the state of the cardiovascular system.

In humans, continuous blood pressure waveform measurements can be collected using an indwelling arterial catheter while fully implanted radiotelemetry devices are capable of remotely collecting blood pressure waveform data from conscious, freely moving research animals (Sand *et al* 2015). Non-invasive monitoring technologies also exist (Li *et al* 2016). In all cases, sampling frequencies can be high (typically 250–1000 Hz or higher) and data collection can take place over long time periods (days to weeks). However, these time series are often irregular, strongly non-stationary and noisy. The classic problem, having collected a large quantity of data, is to derive useful information from it. As Sydney Brenner, a Nobel prize winner, said: ‘We are drowning in a sea of data and starving for knowledge’ (Brenner 2003).

A simple analysis of the data consists of filtering (to remove obvious artefacts), averaging and time-binning and is often presented as discontinuous blocks averaged over a finite period of time. Typically measures such as the maximum, minimum, mean (systolic, diastolic and mean arterial pressure), peak to peak amplitude (pulse pressure) and rate (heart rate) are reported, but this ignores much of the raw waveform data. Alternatively, the data may be analysed in the time domain, transformed into the frequency domain, or analysed using various nonlinear approaches which are often derived from the theory of nonlinear dynamical systems (O’Rourke 2009, Voss *et al* 2009). However these approaches do not have wide clinical uptake, possibly because they require some degree of data post-processing and/or because the clinical interpretation of the analysis is not always clear. This extra burden is not appropriate for the clinical setting.

A common approach to the analysis of time series data is to transform it into the frequency domain using an FFT. This gives information on the various frequencies that are contained in the waveform data, but provides no information about changes occurring in the data at a point in time. Alternatively, a short time Fourier transform can be used on a moving window of data which provides information on changes in frequency at various times, but non-stationarity of the data compromises the frequency resolution (Acharya *et al* 2006).

Methods to obtain a better understanding of the information captured within cardiovascular waveforms have been investigated for a number of decades. Whilst a number of approaches have been taken to quantify and interpret the variability of cardiovascular signals, the associated physiological relevance of these measures has been the subject of much debate and there is still disagreement around this (Parati *et al* 2006). Much attention in this area has focussed on heart rate variability (HRV) which considers variability in the beat-to-beat intervals, which are influenced by both the sympathetic and parasympathetic nervous systems, as well as many other factors (Karim *et al* 2011). This variability was first identified by Hon and Lee in 1963 (Hon and Lee 1963) as a physiological biomarker that can predict foetal distress. It is well established that subtle changes in cardiovascular physiology, as measured by HRV, correlate with many physiological conditions including myocardial infarction, cardiac arrhythmias and renal failure (Acharya *et al* 2006, Karim *et al* 2011) and have been proposed as early clinical markers in sepsis and post-stroke infections (Hon and Lee 1963, Pontet *et al* 2003, Günther *et al* 2012).

From a mathematical perspective, it has long been debated as to whether the heart rate is chaotic or not. In 2009, the journal *Chaos* addressed the question ‘Is the normal heart rate chaotic?’ (Glass 2009). The contributed papers used a variety of methods to address this issue, ranging from deterministic to stochastic. Responses to the question posed include that ‘normal heartbeat series are nonchaotic, nonlinear, and multifractal’ (Baillie *et al* 2009), ‘such a task is actually a difficult problem in the case of heart rates’ (Freitas *et al* 2009) and ‘HRV data are mostly stochastic’ (Hu *et al* 2009).

A Web of Science search for articles on ‘heart rate variability’ or ‘HRV’ gives over 26 000 results, with many thousands in recent years. However, a recent report in 2015 (Sassi *et al* 2015) concluded that ‘The novel approaches to HRV analysis … [have] contributed in the technical understanding of the signal character of NN sequences. On the other hand, their success in developing new clinical tools, such as those for the identification of high-risk patients, has been so far rather limited’ (p 1349). Moreover, the physiological interpretation of the data is often complex and controversy exists regarding the meaning of HRV measures (Billman 2013).

The first step for all HRV analysis is the extraction of beat-to-beat intervals from an ECG or blood pressure signal. There are many available methods for doing this and it has also been recommended that ‘manual editing of the RR data should be performed to a very high standard’ (Cam *et al* 1996, p 364), although the large amount of data currently collected makes this impractical. The vast array of HRV methods then analyse this reduced time series in a wide variety of different ways. However, in extracting the beat-to-beat intervals, the majority of the data that makes up the entire waveform has already been discarded before the start of any analysis. For both ECG and blood pressure signals, various features of the signal have been characterised. These include the PQRST points and various intervals of the ECG waveform or the systolic, mean and diastolic pressures, the augmentation index and the position and morphology of the dichrotic notch and wave reflections on a blood pressure waveform. Subtle changes in the waveform shape can occur in response to normal activity, drug effects or changes in the underlying pathophysiology (O’Rourke 1995) and more information could be gleaned from the signal by detecting such changes in addition to variability in the heart rate.

HRV methods have been thoroughly explored for decades. Given the technological advances in monitoring systems, we consider that it is time to move beyond HRV and to develop a new generation of methods of analysis of physiological data that analyse *all of the data contained within a particular waveform*, not just interval lengths. Our approach is to use attractor reconstruction (using the entire data) to represent the data in a bounded phase space, such that changes in particular features of the waveform can be associated with specific changes in the reconstructed attractor. Clearly, this approach has the potential for extracting much more diagnostic information from the waveform data that is already routinely collected than is possible from an HRV analysis. Further, by using all of the data contained within any particular waveform, there is little requirement for a scientist/clinician to manipulate or process the data which limits the introduction of bias in preclinical and clinical data interpretation.

Attractor reconstruction using delay coordinates was first proposed by Takens (1981) and has since been applied to many types of experimental data including blood pressure data (Narayana Dutt and Krishnan 1999), plethysmographic signals (Deloya Vélez *et al* 2014), respiration (Small *et al* 1999) and EEG time series (Wang *et al* 2010), to name but a few. The advantage of this approach is that a biological signal that is typically visualised as stretched out along the time axis can be represented in a bounded reconstructed phase space. From the reconstructed attractor, various dynamic invariants can be estimated, such as the largest Lyapunov exponent, correlation dimension or entropy (Acharya *et al* 2006, Wang *et al* 2010, Deloya Vélez *et al* 2014). Attractor reconstruction is one of the methods used for HRV analysis but, in this context, it is applied to the reduced RR interval data (Acharya *et al* 2006). In contrast, our approach is to use attractor reconstruction using all of the waveform data. By doing so, we are able to limit the introduction of inadvertent bias and to represent the gradients and contours of the waveform in a manner which allows us to extract extra information from the input signal. This may provide a deeper understanding of physiological or pathological changes within the cardiovascular system that may be missed when focussing on maxima, minima and interval data alone.

In section 2, we describe the four steps of our attractor reconstruction method. We start with a quick overview of the steps and then consider each of them in more detail. In section 3, we analyse some artificial periodic signals composed of piecewise polynomials in order to identify the link between some features of a typical blood pressure signal and properties of our attractor in the reconstructed phase space. Section 4 describes some properties of the attractor that we monitor as a time window moves through the data, while in section 5 we apply our approach to some mouse blood pressure data and show that our method is able to detect changes that HRV does not. We consider more artificial signals in section 6 which have fixed cycle length, and hence no variability in heart rate, and show that variability in the upstroke results in a different attractor from variability in the downstroke. Section 7 contains a brief description of the method applied to other types of physiological signals, including human blood pressure data, which is followed in section 8 by a discussion of this new method, some conclusions and a summary detailing the association between the attractor features and their physiological meaning. All the proofs of the various results stated in the paper are presented in the appendix.

## 2. Attractor reconstruction method

Our aim is to extract diagnostic information from blood pressure data with high sampling frequency, utilising the numerical waveform data in its entirety. We first give an overview of our method and then review each of the steps in more detail.

### 2.1. Overview

Our attractor reconstruction method consists of four fundamental steps, which we now summarise.

**Figure 1.**
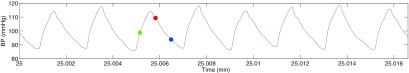
A small sample (1 s) of blood pressure data from a healthy conscious mouse. If the blue dot is *x*(t^*^) for some time point t^*^, then the red dot is $y(t^*)=x(t^*-\tau)$ and the green dot is $z(t^*)=x(t^*-2\tau)$.

1. **Reconstruct an attractor using delay coordinates**
  The first step is to reconstruct an attractor using Takens’ delay coordinates (Takens 1981) for data in a given time window. We choose the embedding dimension to be *n*  =  3 and the time delay *τ* to be one third of the average cycle length of the data in the time window. The reason for these choices is discussed in sections 2.2 and 2.4.
  With an embedding dimension of *n*  =  3, if the signal is $x(t)$ then we define the two new variables
  1 \begin{align*} \newcommand{\e}{{\bf e}} \displaystyle \label{yzdef} y(t)=x(t-\tau), \qquad z(t)=x(t-2\tau) \nonumber \end{align*}
  for a fixed time delay $\tau&gt;0$ (see figure 1). We can then plot the data in the reconstructed phase space as $(x(t), y(t), z(t))$ for all *t* in the given time window.
2. **Remove baseline variation**
  We note that the variables in our reconstructed attractor are all derived from the one signal $x(t)$. If we shift the signal up or down by a constant amount, so that $x(t)\to x(t)+c$ for some $c\in{\bf R}$, then this implies that $y(t)\to y(t)+c$ and $z(t)\to z(t)+c$ also. In the phase space, the shift in our signal $x(t)$ implies that $(x(t), y(t), z(t))\to(x(t)+c, y(t)+c, z(t)+c)=(x(t), y(t), z(t))+c(1, 1, 1)$ which corresponds to a shift in the reconstructed phase space in the direction of the vector $(1, 1, 1)$. To eliminate the effect of a constant vertical translation, we project our three-dimensional attractor onto a plane that is perpendicular to the vector $(1, 1, 1)$. Thus, we define the new variables
  2 \begin{align*} \newcommand{\e}{{\bf e}} \displaystyle \label{uvw} u=\frac{1}{3}(x+y+z),~~v=\frac{1}{\sqrt{6}}(x+y-2z),~~w=\frac{1}{\sqrt{2}}(x-y). \nonumber \end{align*}
  It is easily verified that a constant vertical shift in the signal $x(t)$ implies that $u(t)\to u(t)+c$ but that the coordinates *v* and *w* are invariant. Thus, projecting the attractor onto the $(v, w)$ plane has the effect of removing such vertical translations.
3. **Construct a density**
  One of the problems of an attractor plotted in phase space is that it can become a blur of lines with little detail visible. In order to avoid this, we derive a density from our reconstructed attractor that has been projected onto the $(v, w)$ plane. The density provides more information regarding the attractor since it can distinguish between high density regions which are visited frequently, and low density regions which indicate infrequent variations.
4. **Generate time traces of attractor measures**
  For the fourth and final step of our approach, we extract a quantity of interest from the density in the $(v, w)$ plane that has been derived using a given time window of data. As a simple example, we could determine the maximum value of the density. Repeating this process as the time window is moved through the data gives a time trace of the maximum density.
  The purpose of this approach is to use a collection of these time traces that have been obtained from various features of the density in order to provide diagnostic information regarding the signal.

We now review each of these steps in detail together with an example to illustrate the method. The data that we use is a single stream of blood pressure data sampled at 1000 Hz that has been collected from a healthy, conscious mouse using an implanted radiotelemetry device. More details of the data collection are given in section 5.

### 2.2. Attractor reconstruction using delay coordinates

When blood pressure data are viewed over long time intervals all that can be readily observed is the general pattern of the rise and fall of the average blood pressure and some indication of changes in the pulse pressure (amplitude), but little else. However, there is great variety within this signal as there are many factors that influence the blood pressure in a conscious animal, including the sympathetic and parasympathetic nervous systems, respiratory system and motor activity (Karim *et al* 2011). A full mathematical model of blood pressure that incorporated all of these factors would be very complicated and high-dimensional.

When (numerically) solving a system of nonlinear differential equations, the solutions can be plotted as a function of time, but it is often not possible to see any structure in the solutions in this way. A more useful representation is to plot the trajectory in the phase space as the attractor is then contained in a bounded region. Even for chaotic systems, such as the Lorenz equations, some structure can be seen in the attractor when it is plotted in the phase space (Sparrow 1982).

When working with experimental data, a plot of the trajectory in the phase space would require each of the variables in the model equations to be measured. In most cases, measuring all such quantities is simply not possible. In many other cases, there are no model equations and so it is not even clear what should be measured. A commonly occurring situation is that a single quantity, such as blood pressure, is measured experimentally over a given time period. With only a single signal, it would seem that there is not enough information available to generate a plot of the trajectory in a phase space.

In 1981, Takens (1981) considered this problem of deriving information regarding a dynamical system from a single continuous observed variable. He showed that an attractor can be reconstructed in an *n*-dimensional ‘phase space’ from a single signal $x(t)$ by using a vector of delay coordinates

\begin{align*} \newcommand{\e}{{\bf e}} \displaystyle [x(t), x(t-\tau), x(t-2\tau),\ldots,x(t-(n-1)\tau)] \nonumber \end{align*}

where $\tau&gt;0$ is a fixed delay and $n\geqslant 2$ is the embedding dimension. This method has since been widely used to reconstruct chaotic attractors, including the familiar Lorenz attractor (Pecora *et al* 2007). It can be seen that the reconstructed attractor is qualitatively similar to the original (Abarbanel *et al* 1993).

While Takens’ theorem considers a *C*^2^ measurement function of the flow of a vector field, his approach has also been applied in many circumstances to a single stream of experimental data. If the data are obtained by sampling an underlying *C*^2^ function, then working with the data is essentially a discretised version of Takens’ approach. Of course much experimental data is subject to noise, in which case Takens’ method strictly does not apply. However, it is often applied where there is a certain amount of noise and generally works well provided that the noise is sufficiently small. Takens’ method has been applied to many types of experimental data including the analysis of blood pressure data (Narayana Dutt and Krishnan 1999), plethysmographic signals (Deloya Vélez *et al* 2014), respiration (Small *et al* 1999) and EEG time series (Wang *et al* 2010), to name but a few.

From a practical point of view, the two key choices to be made when using Takens’ delay coordinates to reconstruct an attractor are (i) the choice of the embedding dimension *n*, and (ii) the value of the time delay *τ* to be used. With regard to the embedding dimension, Takens showed that for an *m*-dimensional manifold, 2*m*  +  1 delay coordinates are sufficient to give a diffeomorphic reconstruction, although a lower embedding dimension also works in many cases. As discussed previously, the dimension of the model or of the attractor is generally not known, and so this theoretically interesting result is of little practical assistance. Various methods have been proposed for determining a minimum dimension for the reconstructed attractor including a singular value analysis and the method of false nearest neighbours (Kennel *et al* 1992, Abarbanel *et al* 1993, Kennel and Abarbanel 2002).

The other variable to be chosen is the time delay *τ*. Theoretically, there are no restrictions on *τ* (except that it should be positive). If *τ* is very small, then there will be only a small difference between the variables, and so the trajectory will always lie close to the axis in the phase space given by $x_1=x_2=\ldots=x_n$, where $x_i(t)=x(t-(i-1)\tau)$, $i=1, \ldots, n$. On the other hand, if *τ* is chosen to be very large, then there may be little correlation between each of the variables. So *τ* should be chosen in a middle range, avoiding these two extremes. A common method for choosing the value of *τ* is based on minimising mutual information (Fraser and Swinney 1986, Abarbanel *et al* 1993).

A continuity statistic has been proposed as a measure to determine both the optimal time delay and embedding dimension simultaneously (Pecora *et al* 2007).

When Takens’ method is applied to data, the traditional approach is to find the optimal embedding dimension and time delay using one of the methods described above, and then to generate the reconstructed attractor using these optimal parameters. From this attractor, various dynamic invariants are estimated, such as the largest Lyapunov exponent, correlation dimension or entropy (Acharya *et al* 2006, Wang *et al* 2010, Deloya Vélez *et al* 2014).

The motivation for Takens’ method was to reconstruct a faithful attractor in phase space. Our aim is fundamentally different from this and so we do not use the standard methods for choosing the embedding dimension *n* and time delay *τ*. Our aim is to use properties of a reconstructed attractor to provide information regarding key features of the data, so that dynamic changes in the data can be detected from dynamic changes in the attractor. To keep the method as simple as possible, and to be able to easily visualise the reconstructed attractor, we choose an embedding dimension of *n*  =  3. Given a (continuous) signal $x(t)$, two extra variables $y(t)$ and $z(t)$ are defined as in (1). We can then plot the data in a given time window in the three-dimensional $(x, y, z)$ phase space.

**Figure 2.**
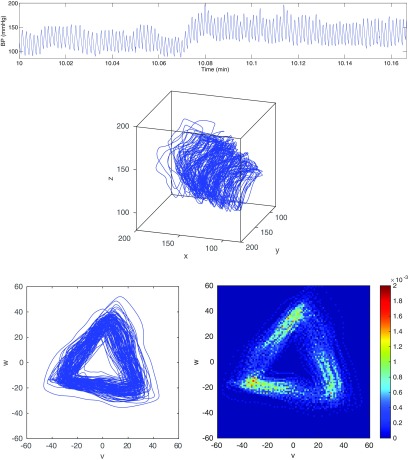
Top: A 10 s sample of blood pressure data from a healthy, conscious mouse. Middle: A trajectory in the three-dimensional reconstructed phase space using $\tau=30$ ms. Bottom left: A projection of the trajectory onto the plane orthogonal to the *x*  =  *y*  =  *z* axis. Bottom right: The trajectory turned into a density. Bottom images © 2014 IEEE. Reprinted, with permission, from Aston *et al* 2014. CC BY 2.5.

A ten second sample of blood pressure data from a healthy, conscious mouse is shown in figure 2 (top). The attractor in a three-dimensional reconstructed phase space for $\tau=30$ ms is also shown (middle). The choice of the time delay *τ* will be discussed in section 2.4.

### 2.3. Removal of baseline variation

When the blood pressure data are viewed over a long time interval, the individual oscillations can no longer be distinguished and the only information that is readily observable is the vertical motion of the average blood pressure which will vary depending on whether the animal is resting, active, eating, sleeping, etc. It is this natural variation, resulting in a non-stationary signal, which makes it difficult to analyse the frequencies using, for example, an FFT (Acharya *et al* 2006).

Many methods have been proposed in the literature for removing baseline variation, particularly from ECG signals. These generally consist of either approximating the baseline, which is then subtracted from the signal, or of filtering the data to remove the low frequencies. Meyer *et al* (1977) determined the baseline in an ECG signal by using an averaged point in each PR segment and joining up these points using a cubic spline. Adaptive filtering was proposed by Laguna *et al* (1992) to remove baseline wander while Zhang (2005) used a discrete wavelet transform. A more recent method involves solving a constrained convex optimisation problem based on the quadratic variation of the signal (Fasano *et al* 2011). A variety of methods for removing baseline variation from an ECG signal was reviewed by Kaur *et al* (2011) who concluded that IIR zero phase filtering was the best of the methods considered. Heart rate variability (HRV) methods consider cycle lengths derived from the signal (Acharya *et al* 2006). These intervals are independent of any vertical motion in the signal and so HRV methods also implicitly eliminate baseline variation.

We are using blood pressure as our cardiovascular physiological signal. In order to extract information regarding the waveform shape, it is useful to remove baseline variation. However, we do this in a different way from the current methods in the literature. We note that the variables in our reconstructed attractor are all derived from the one signal $x(t)$. So if we shift the signal up or down by a constant amount, so that $x(t)\to x(t)+c$ for some $c\in{\bf R}$, then this implies that $(x(t), y(t), z(t))\to(x(t), y(t), z(t))+c(1, 1, 1)$, as shown in section 2.1. This corresponds to a shift in our reconstructed phase space in the direction of the vector $(1, 1, 1)$. In order to remove the vertical variation in the signal, we define a new coordinate system which consists of the vector $(1, 1, 1)$ and two further vectors that are orthogonal to this one (and to each other). Normalising these vectors gives an orthonormal basis for our three-dimensional phase space. We then project the trajectory in our three-dimensional phase space onto the two-dimensional plane orthogonal to the vector $(1, 1, 1)$ and this has the effect of factoring out the vertical variation in the signal.

Physiologically, this approach therefore ignores the magnitude of the maxima and minima of the waveform, namely the absolute systolic and diastolic pressures. The projection onto the plane factors out the baseline variation of the signal which ensures that changes over time in the shape and frequency of the waveform can be described. This now provides a unique method to exclusively quantify waveform morphology and variability changes which previous studies have shown may contain important diagnostic information (O’Rourke *et al* 2016).

We define the line through the origin of the phase space in the direction of the vector $(1, 1, 1)$, namely the line on which *x*  =  *y*  =  *z*, as the *central axis* of the phase space. A unit vector in the direction of the central axis is given by $ \newcommand{\vv}{{\bf v}} \vv_1=(1, 1, 1){\hspace{0pt}}^T/\sqrt{3}$. The remaining two basis vectors must be orthogonal to this one and to each other and we choose the (unit) vectors $ \newcommand{\vv}{{\bf v}} \vv_2=(1, 1, -2){\hspace{0pt}}^T/\sqrt{6}$ and $ \newcommand{\vv}{{\bf v}} \vv_3=(1, -1, 0){\hspace{0pt}}^T/\sqrt{2}$. Thus, the matrix *M* which has columns $ \newcommand{\vv}{{\bf v}} \vv_1$, $ \newcommand{\vv}{{\bf v}} \vv_2$ and $ \newcommand{\vv}{{\bf v}} \vv_3$ is an orthogonal matrix. If we have coordinates $(u, v, w)$ with respect to the new basis vectors, then the old and new coordinates are related by

\begin{align*} \newcommand{\vv}{{\bf v}} \newcommand{\e}{{\bf e}} \displaystyle \left[\begin{array}{@{}c@{}}x \nonumber \\y \nonumber \\z\end{array}\right]=u\vv_1+v\vv_2+w\vv_3 \nonumber \end{align*}

or equivalently

\begin{align*} \newcommand{\uu}{{\bf u}} \newcommand{\x}{{\bf x}} \newcommand{\e}{{\bf e}} \displaystyle \x=M\uu \nonumber \end{align*}

where $ \newcommand{\x}{{\bf x}} \x=(x, y, z){\hspace{0pt}}^T$ and $ \newcommand{\uu}{{\bf u}} \uu=(u, v, w){\hspace{0pt}}^T$. Thus, the new coordinates are defined by

\begin{align*} \newcommand{\uu}{{\bf u}} \newcommand{\x}{{\bf x}} \newcommand{\e}{{\bf e}} \displaystyle \uu=M^T\x \nonumber \end{align*}

since *M* is an orthogonal matrix, or equivalently

\begin{align*} \newcommand{\x}{{\bf x}} \newcommand{\vv}{{\bf v}} \newcommand{\e}{{\bf e}} \displaystyle u&amp;=\vv_1^T\x~=~\frac{1}{\sqrt{3}}(x+y+z)\nonumber \\ v&amp;=\vv_2^T\x~=~\frac{1}{\sqrt{6}}(x+y-2z)\nonumber \\ w&amp;=\vv_3^T\x~=~\frac{1}{\sqrt{2}}(x-y). \nonumber \end{align*}

From this, we see that *u* is almost the mean of the three original variables *x*, *y* and *z*. It is more natural to redefine *u* to be the mean, and so we will work with the three variables *u*, *v* and *w* that we defined earlier in (2). It follows from these definitions that

3 \begin{align*} \newcommand{\e}{{\bf e}} \displaystyle \label{xuvw} x=u+\frac{1}{\sqrt{6}}v+\frac{1}{\sqrt{2}}w \nonumber \end{align*}

and so these new variables can also be considered as a decomposition of the original signal into three component parts.

**Figure 3.**
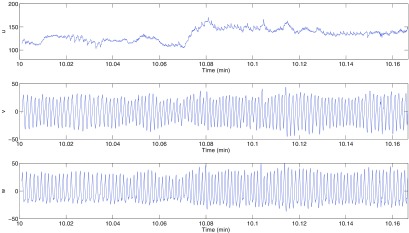
Trajectories derived from the blood pressure data shown in figure 2 (top). Plots are shown for the variables *u* (top), *v* (middle) and *w* (bottom).

It is easily verified that if $x(t)\to x(t)+c$ for some $c\in{\bf R}$, then $(u(t), v(t), w(t))\to(u(t)+c, v(t), w(t))$. Thus the new variable $u(t)$ captures the vertical motion of the blood pressure signal, but the other two variables $v(t)$ and $w(t)$ are not affected by this motion, and so can be used to derive other information from the signal. This is illustrated in figure 3 for the window of data shown in figure 2 (top). The variable *u* (figure 3 (top)) has clearly picked up the trend in the data with little oscillation while *v* and *w* (figure 3 (middle, bottom)) show no sign of the baseline variation and appear to have approximately zero mean.

Clearly the new variables *v* and *w* are the coordinates of a point in the three-dimensional phase space projected onto the plane orthogonal to the central axis *x*  =  *y*  =  *z*. The trajectory in the three-dimensional phase space in figure 2 (middle) projected onto this plane is shown in figure 2 (bottom left), from which it can be seen that all the variability in the three-dimensional attractor in the direction of the central axis has been removed.

Using the definition of *u* given in (2), we also see that there is a close relationship between the means of *x* and *u* over a given time period. We define the mean of the function $x(t)$ over the time interval $[t^*, t^*+L]$ by

\begin{align*} \newcommand{\e}{{\bf e}} \displaystyle \bar x=\frac{1}{L}\int_{t^*}^{t^*+L}x(t)~{\rm d}t. \nonumber \end{align*}

We then have the following result.

Lemma 2.1. If $x(t)$ is a continuous function on the interval $I=[t^*-2\tau, t^*+L]$ then

\begin{align*} \newcommand{\e}{{\bf e}} \displaystyle \vert \bar u-\bar x\vert \leqslant(M-m)\left(\frac{\tau}{L}\right),~~ \vert \bar v\vert \leqslant\frac{5}{\sqrt{6}}(M-m)\left(\frac{\tau}{L}\right),~~ \vert \bar w\vert \leqslant\frac{1}{\sqrt{2}}(M-m)\left(\frac{\tau}{L}\right)\nonumber \end{align*}

where

\begin{align*} \newcommand{\e}{{\bf e}} \displaystyle M=\max_{t\in I}x(t),~~m=\min_{t\in I}x(t)\nonumber \end{align*}

A similar result also holds if the mean of the continuous functions is replaced by the mean of discrete data points. We note from the proof (given in the appendix) that this result could also be expressed in terms of the maxima and minima of $x(t)$ over the two much smaller intervals $[t^*-2\tau, t^*]$ and $[t^*+L-2\tau, t^*+L]$.

It follows from this result that if the time delay is small compared to the window length ($\tau\ll L$), then the means of *x* and *u* are very similar and the variables *v* and *w* have mean which is very close to zero.

The basis of the analysis above consisted of removing constant translations in the vertical direction. Baseline variation for a physiological signal, on the other hand, does not consist of a vertical translation. However, by considering a Fourier transform of the new variables, we can see that the variable *u* retains the low frequency component of the signal, which is reduced in the *v* and *w* variables.

#### 2.3.1. Fourier transforms

We now consider the Fourier transforms of the variables *u*, *v* and *w*, and consider how these relate to the Fourier transform of the signal *x*. We recall that if $s\in L^1({\bf R})$, then the Fourier transform is defined by

\begin{align*} \newcommand{\x}{{\bf x}} \newcommand{\e}{{\bf e}} \displaystyle \hat s(\xi)={{\mathcal F}}[s(t)]=\int_{-\infty}^\infty s(t){\rm e}^{-2\pi {\rm i}\xi t}~{\rm d}t. \nonumber \end{align*}

Of course our physiological signal is not defined for all $t\in{\bf R}$ (and if it was, it would not be in $L^1({\bf R})$) and so we consider only the signal in a finite window. If $x(t)$ is our continuous blood pressure signal, then we define

\begin{align*} \newcommand{\e}{{\bf e}} \displaystyle X(t)=x(t)r_L(t-t_0) \nonumber \end{align*}

where *r*_*L*_(*t*) is a rectangular window function of height one centred on *t*  =  0 and of width *L*. Thus, $X(t)$ is given by the signal for $t\in[t_0-L/2, t_0+L/2]$ and zero elsewhere. Since $x(t)$ is continuous, then $X(t)$ is a piecewise continuous function with two finite discontinuities and compact support and so the Fourier transform exists (Bracewell 2000). The convolution theorem for the Fourier transform (Pinsky 2002) implies that

\begin{align*} \newcommand{\x}{{\bf x}} \newcommand{\e}{{\bf e}} \displaystyle \hat X(\xi)=\hat x(\xi)\hat r_L(\xi) \nonumber \end{align*}

where $ \newcommand{\x}{{\bf x}} \hat r_L(\xi)$, the Fourier transform of the rectangular window function, is a sinc function (Smith 1997). Thus, taking a finite window of data results in an initial filtering of the spectrum. However, we are interested in the spectrum of the new variables *u*, *v* and *w*, derived from the finite data segment $X(t)$, in relation to the Fourier transform $ \newcommand{\x}{{\bf x}} \hat X(\xi)$ of $X(t)$.

Theorem 2.2. The Fourier transforms of the variables *u*, *v* and *w* are given by

4 \begin{align*} \newcommand{\x}{{\bf x}} \newcommand{\e}{{\bf e}} \displaystyle \hat u(\xi)&amp;=\frac{1}{3}\left(1+{\rm e}^{-2\pi {\rm i}\xi\tau}+{\rm e}^{-4\pi {\rm i}\xi\tau}\right)\hat X(\xi)\nonumber \\ &amp;=\frac{1}{3}(1+2\cos(2\pi\xi\tau)){\rm e}^{-2\pi {\rm i}\xi\tau}\hat X(\xi)\label{uhat} \nonumber \end{align*}

5 \begin{align*} \newcommand{\x}{{\bf x}} \newcommand{\e}{{\bf e}} \displaystyle \hat v(\xi) = \frac{1}{\sqrt{6}}\left(1+{\rm e}^{-2\pi {\rm i}\xi\tau}-2{\rm e}^{-4\pi {\rm i}\xi\tau}\right)\hat X(\xi)\label{vhat} \nonumber \end{align*}

6 \begin{align*} \newcommand{\x}{{\bf x}} \newcommand{\e}{{\bf e}} \displaystyle \hat w(\xi) = \frac{1}{\sqrt{2}}\left(1-{\rm e}^{-2\pi {\rm i}\xi\tau}\right)\hat X(\xi)\label{what} \nonumber \end{align*}

Taking the Fourier transform of (3) and using linearity implies that

7 \begin{align*} \newcommand{\x}{{\bf x}} \newcommand{\e}{{\bf e}} \displaystyle \label{ftdecomp} \hat X(\xi)=\hat u(\xi)+\frac{1}{\sqrt{6}}\hat v(\xi)+\frac{1}{\sqrt{2}}\hat w(\xi) \nonumber \end{align*}

and so the Fourier transforms of *u*, *v* and *w* also provide a decomposition of the Fourier transform of *X*. It is easily seen that the Fourier transforms given in (4)–(6) satisfy (7).

Taking the modulus squared of (4)–(6) gives

8 \begin{align*} \newcommand{\x}{{\bf x}} \newcommand{\e}{{\bf e}} \displaystyle \vert \hat u(\xi)\vert ^2 = \frac{1}{9}\vert 1+2\cos(2\pi\xi\tau)\vert ^2~\vert \hat X(\xi)\vert ^2\label{uhatmod} \nonumber \end{align*}

9 \begin{align*} \newcommand{\x}{{\bf x}} \newcommand{\e}{{\bf e}} \displaystyle \vert \hat v(\xi)\vert ^2 = \frac{1}{3}(5+4\cos(2\pi\xi\tau))(1-\cos(2\pi\xi\tau))\vert \hat X(\xi)\vert ^2\label{vhatmod} \nonumber \end{align*}

10 \begin{align*} \newcommand{\x}{{\bf x}} \newcommand{\e}{{\bf e}} \displaystyle \vert \hat w(\xi)\vert ^2 = (1-\cos(2\pi\xi\tau))\vert \hat X(\xi)\vert ^2.\label{whatmod} \nonumber \end{align*}

From (8)–(10), we can see that *u*, *v* and *w* have the effect of filtering the power spectrum $ \newcommand{\x}{{\bf x}} \vert \hat X(\xi)\vert ^2$ of *X*. The three frequency response functions are given by

**Figure 4.**
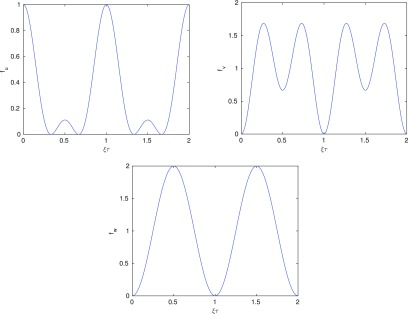
The frequency response functions *f*_*u*_ (top left), *f*_*v*_ (top right) and *f*_*w*_ (bottom).

\begin{align*} \newcommand{\x}{{\bf x}} \newcommand{\e}{{\bf e}} \displaystyle f_u(\xi\tau)&amp;=\frac{1}{9}\vert 1+2\cos(2\pi\xi\tau)\vert ^2\nonumber \\ f_v(\xi\tau)&amp;=\frac{1}{3}(5+4\cos(2\pi\xi\tau))(1-\cos(2\pi\xi\tau))\nonumber \\ f_w(\xi\tau)&amp;=1-\cos(2\pi\xi\tau) \nonumber \end{align*}

and are shown in figure 4.

Since *f*_*u*_(0)  =  1, clearly *u* retains the lowest frequencies from *X* and so includes the low frequency baseline variation. However, $f_v(0)=f_w(0)=0$ (and $f_v'(0)=f_w'(0)=0$) and so these lowest frequencies have been filtered out from *v* and *w*, as we expect.

If the signal is approximately periodic with average cycle length *T*, then the power spectrum will have peaks close to $ \newcommand{\x}{{\bf x}} \xi=k/T$, $k=0, 1, 2,\,\,\! \ldots$ In section 2.4, we choose *τ* to be either one third or two thirds of the average cycle length. If we choose $\tau=T/3$, then $ \newcommand{\x}{{\bf x}} \xi\tau=k/3$. We note that $ \newcommand{\x}{{\bf x}} f_u(\xi\tau)=0$ for $ \newcommand{\x}{{\bf x}} \xi\tau=n/3, 2n/3$, $n=1, 2, \ldots$ (which can also be seen from figure 4 (top left)) and so *f*_*u*_ filters out many, but not all, of the periodic components in the signal in this case. However, *f*_*v*_ and *f*_*w*_ clearly amplify these peaks and so contain more of the dominant periodic component of the signal. Similar results hold when $\tau=2T/3$.

Clearly these results are for the power spectrum of the continuous signal. However, data sampled at (equally spaced) time points in a time window can be analysed using a discrete Fourier transform (DFT), and similar results also apply.

This can be seen in figure 3 (top) where *τ* was chosen as one third of the average cycle length and we see that *u* essentially picks up the baseline variation in the signal with a small amplitude higher frequency superimposed oscillation, whereas *v* and *w* have very little baseline variation and are much closer to periodicity than the original signal (see figure 3 (middle, bottom)).

### 2.4. Choice of the time delay

To motivate our choice of the time delay parameter $\tau&gt;0$, we consider a sine wave with period 1 given by

**Figure 5.**
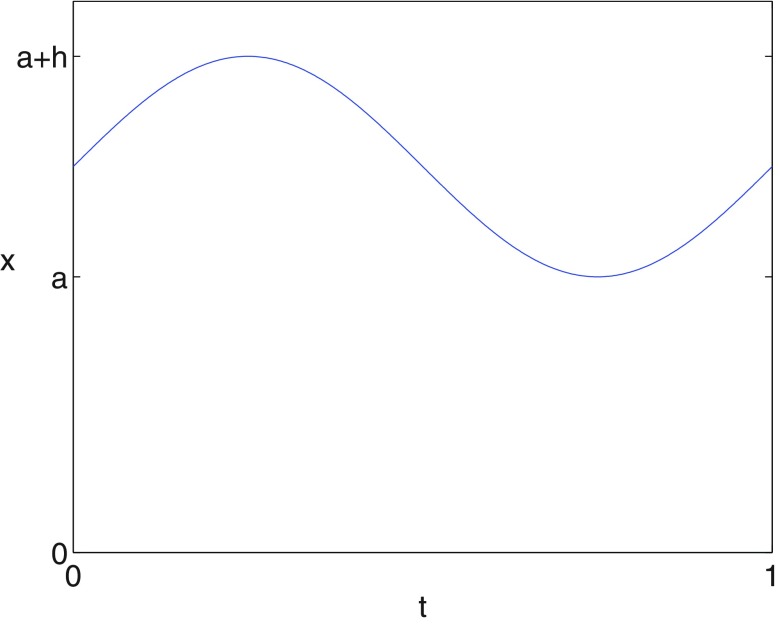
A sinusoidal signal $x(t)=a+(h/2)(1+\sin(2\pi t))$.

\begin{align*} \newcommand{\e}{{\bf e}} \displaystyle x(t)=a+\frac{h}{2}(1+\sin(2\pi t))\nonumber \end{align*}

which is shown in figure 5.

For a periodic signal with period *T*, we note that if $\tau=0$ or $\tau=T$ then $x(t)=y(t)=z(t)$ and so the orbit simply moves up and down the central axis, which corresponds to the point at the origin of the $(v, w)$ plane. Thus, we make the natural assumption that $\tau\in(0, T)$. We also note that if *x* is periodic, then the orbit in the three-dimensional $(x, y, z)$ phase space as well as the orbit in the two-dimensional projection $(v, w)$ must be a closed curve. The shape of this closed orbit for varying values of *τ* is shown as an animation (see supplementary material (stacks.iop.org/PM/39/024001/mmedia)). We now describe some of the changes that are seen in this animation.

For the sine wave, our extra phase space variables *y* and *z* are

\begin{align*} \newcommand{\e}{{\bf e}} \displaystyle y(t)&amp;=a+\frac{h}{2}(1+\sin(2\pi(t-\tau)))\nonumber \\ z(t)&amp;=a+\frac{h}{2}(1+\sin(2\pi(t-2\tau))). \nonumber \end{align*}

From the definition of our transformed variables *v* and *w* given by (2), we then have

\begin{align*} \newcommand{\e}{{\bf e}} \displaystyle v(t)&amp;=\frac{h}{2\sqrt{6}}[\sin(2\pi t)+\sin(2\pi(t-\tau))-2\sin(2\pi(t-2\tau))]\nonumber \\ &amp;=\frac{h}{2\sqrt{6}}[(1-\cos(2\pi\tau))(4\cos(2\pi\tau)+3)\sin(2\pi t)+\sin(2\pi\tau)(4\cos(2\pi\tau)-1)\cos(2\pi t)]\nonumber \\ w(t)&amp;=\frac{h}{2\sqrt{2}}[\sin(2\pi t)-\sin(2\pi(t-\tau))]\nonumber \\ &amp;=\frac{h}{2\sqrt{2}}[(1-\cos(2\pi\tau))\sin(2\pi t)+\sin(2\pi\tau)\cos(2\pi t)]. \nonumber \end{align*}

The parameter *a* in the definition of the periodic signal *x* represents the vertical position of the signal and since our new variables *v* and *w* are independent of vertical translations, then they must be independent of the parameter *a*, which is of course the case.

Considering $\sin(2\pi t)$ and $\cos(2\pi t)$ as independent variables, we can write these equations as

11 \begin{align*} \newcommand{\w}{{\bf w}} \newcommand{\e}{{\bf e}} \displaystyle \label{twoequations} \left(\begin{array}{@{}c@{}}v \nonumber \\w\end{array}\right)= \left(\begin{array}{@{}cc@{}}a &amp; b \nonumber \\c &amp;d\end{array}\right) \left(\begin{array}{@{}c@{}}\sin(2\pi t) \nonumber \\ \cos(2\pi t)\end{array}\right) \nonumber \end{align*}

where

\begin{align*} \newcommand{\e}{{\bf e}} \displaystyle a&amp;=\frac{h}{2\sqrt{6}}(1-\cos(2\pi\tau))(4\cos(2\pi\tau)+3)\nonumber \\ b&amp;=\frac{h}{2\sqrt{6}}\sin(2\pi\tau)(4\cos(2\pi\tau)-1)\nonumber \\ c&amp;=\frac{h}{2\sqrt{2}}(1-\cos(2\pi\tau))\nonumber \\ d&amp;=\frac{h}{2\sqrt{2}}\sin(2\pi\tau). \nonumber \end{align*}

If *ad*  −  *bc*  =  0 then the $2\times 2$ matrix is singular and eliminating *t* from these equations gives

12 \begin{align*} \newcommand{\e}{{\bf e}} \displaystyle \label{line} v=\frac{a}{c}w \nonumber \end{align*}

which is always well defined since $c\neq 0$ for all $\tau\in(0, 1)$. In this case, the closed curve in the $(v, w)$ plane must occur along the straight line through the origin given by (12). We note that

\begin{align*} \newcommand{\e}{{\bf e}} \displaystyle ad-bc=\frac{h^2}{2\sqrt{3}}\sin(2\pi\tau)(1-\cos(2\pi\tau))\nonumber \end{align*}

and so *ad*  −  *bc*  =  0 when either $\sin(2\pi\tau)=0$ or $\cos(2\pi\tau)=1$. The only solution of either of these equations for $\tau\in(0, 1)$ is $\tau=1/2$. In this case, the closed orbit lies on the line $v=-w/\sqrt{3}$.

Thus, for all $\tau\in(0, 1)\backslash\{\frac{1}{2}\}$, we have $ad-bc\neq 0$ and so equation (11) can be solved uniquely for $\sin(2\pi t)$ and $\cos(2\pi t)$. Substituting the solution into the trigonometric identity $\cos^2(2\pi t)+\sin^2(2\pi t)=1$ gives the quadratic form

\begin{align*} \newcommand{\e}{{\bf e}} \displaystyle (c^2+d^2)x^2-2(ac+bd)xy+(a^2+b^2)y^2-(ad-bc)^2=0. \nonumber \end{align*}

This equation defines one of the conic sections and it is easily verified that this is generally an ellipse, except in the special case when

\begin{align*} \newcommand{\e}{{\bf e}} \displaystyle ac+bd=0~{\rm and}~c^2+d^2=a^2+b^2\nonumber \end{align*}

in which case the solution is a circle. Expressing these two equations in terms of *τ* and simplifying gives the same equation in both cases, namely

\begin{align*} \newcommand{\e}{{\bf e}} \displaystyle (2\cos(2\pi\tau)+1)(\cos(2\pi\tau)-1)=0. \nonumber \end{align*}

The solutions of this equation with $\tau\in(0, 1)$ are $\tau=1/3, 2/3$.

Our approach to choosing the time delay *τ* is to make the projected attractor in the $(v, w)$ plane ‘as uniform as possible’. The reason for this geometric criterion is to make any changes from the uniform case more visible and hence easier to detect and quantify. For this simple example, the requirement for the attractor to be as uniform as possible suggests that we should choose one of the two *τ* values that result in a circular orbit in the $(v, w)$ plane, namely $\tau=1/3$ or $\tau=2/3$.

To complete this example, we note that when $\tau=1/3$, we have

\begin{align*} \newcommand{\e}{{\bf e}} \displaystyle v(t)=\frac{\sqrt{3}h}{2\sqrt{2}}\cos\left(\frac{5\pi}{6}-2\pi t\right),~~ w(t)=\frac{\sqrt{3}h}{2\sqrt{2}}\sin\left(\frac{5\pi}{6}-2\pi t\right)\nonumber \end{align*}

and so the circular orbit in the $(v, w)$ plane has radius $r=\sqrt{3}h/\left(2\sqrt{2}\right)$, which is proportional to the wave amplitude *h*, and the motion is uniform in a clockwise direction. When $\tau=2/3$, the circular orbit has the same radius but the motion is uniform in an anticlockwise direction. Moreover, in both cases, $u(t)=a+h/2$ is constant and is the mean of the signal.

Of course, we have so far only considered the most trivial periodic example. We next retain the assumptions that the signal $x(t)$ is continuous and has period 1, but make no additional assumptions. In this case, some aspects of the above example carry over. In particular the values of $\tau=1/3$, $\tau=1/2$ and $\tau=2/3$ are significant in this case also, as is shown in the following result.

Theorem 2.3. Assume that $x(t)$ is continuous and periodic with period 1.

(i) If $\tau=1/3$, then the closed trajectory in the $(v, w)$ plane has ${\bf Z}_3$ symmetry generated by a rotation of $2\pi/3$ about the origin and the trajectory goes in a clockwise direction. In addition, $u(t)$ has period 1/3.
(ii) If $\tau=1/2$, then the trajectory in the $(v, w)$ plane lies on the line $v=-w/\sqrt{3}$.
(iii) If $\tau=2/3$, then the trajectory in the $(v, w)$ plane also has ${\bf Z}_3$ symmetry generated by a rotation of $2\pi/3$ about the origin and is obtained from the trajectory with $\tau=1/3$ by reflecting it in the *v* axis ($w\to -w$). The trajectory goes in an anticlockwise direction. In addition, $u(t)$ is the same as for $\tau=1/3$ and so again has period 1/3.

We note that the orbit in the $(v, w)$ plane collapses onto a line when $\tau=1/2$ for any periodic signal. With our criterion for choosing *τ* that the attractor should be as uniform as possible, this is clearly the worst possible choice. For $\tau=1/3$ and $\tau=2/3$, the sine wave signal gives rise to a circular orbit in the $(v, w)$ plane which has arbitrary rotational symmetry as well as a reflectional symmetry (i.e. O(2) symmetry). For a more general periodic signal, the reflectional symmetry is lost and only a ${\bf Z}_3$ rotational symmetry is retained. However, this rotational symmetry still gives some structure and a degree of uniformity to the orbit, and so the best choices of *τ* are clearly $\tau=1/3$ or $\tau=2/3$ for a signal with period one.

Returning to our blood pressure signal, this of course is not strictly periodic, but could be described as ‘approximately periodic’ due to the cyclic nature of the heartbeats. Thus, for a given time window of data, we determine an average cycle length for that window of data and choose *τ* to be either one third or two thirds of that quantity. This will result in an attractor in the $(v, w)$ plane with approximate threefold rotational symmetry. We will usually choose the ‘short range’ *τ* as one third of the average cycle length, which gives the three points quite close together. For the ‘long range’ *τ* of two thirds of the average cycle length, the three points will always be sampling from two different cycles since the first and last points are four thirds of the average cycle length apart.

A common approach for finding the dominant frequency in a time series is to use an FFT. However, the blood pressure data are a non-stationary time series which often results in poor resolution of the various frequencies in the data (Ivanov *et al* 2004). It can be seen from the FFTs of blood pressure data in Christie *et al* (2013) that the peaks in the frequency spectrum are very broad, which causes problems when trying to accurately estimate the dominant frequency.

One standard method of finding an average cycle length from approximately periodic data is autocorrelation (Oppenheim 2010). For discrete data $y_1, \ldots, y_n$, if $\bar y$ is the sample mean then we define

\begin{align*} \newcommand{\e}{{\bf e}} \displaystyle r(T)=\frac{1}{n}\sum_{i=T+1}^{n}(y_{i-T}-\bar y)(y_i-\bar y)\nonumber \end{align*}

which is often normalised by the sample variance $r(0)$. Thus, values of the function

\begin{align*} \newcommand{\e}{{\bf e}} \displaystyle R(T)=\frac{r(T)}{r(0)}\nonumber \end{align*}

are evaluated for a range of *T* values. Clearly $R(0)=1$ and so the value of *T*  >  0 corresponding to the highest local maximum is considered to be the average cycle length.

Restricting to the time window $t\in[t^*, t^*+L]$, an alternative approach, which we will use, is to find the average cycle length *T* by minimising $\Vert x(t)-x(t-T)\Vert $ for some appropriate norm, where the norm is evaluated for $t\in[t^*+T, t^*+L]$. Clearly if $x(t)$ is periodic with period T^*^, then the norm will be zero for *T*  =  T^*^ (or any multiple of T^*^). The norm is also clearly zero when *T*  =  0 and increases for increasing *T*. For approximately periodic data, we define the average cycle length to be the value of *T*  >  0 corresponding to the first local minimum.

In order to remove the scale of the data, and any dependence on the window length, we instead minimise

13 \begin{align*} \newcommand{\e}{{\bf e}} \displaystyle \label{fofT} f(T)=\frac{\Vert x(t)-x(t-T)\Vert}{\Vert \bar x {\bf 1}\Vert}=\frac{\Vert x(t)-x(t-T)\Vert}{\bar x\Vert {\bf 1}\Vert} \nonumber \end{align*}

where **1** is a vector with all entries having the value 1, and the same length as the number of data points in the window of data, and $\bar x&gt;0$ is some fixed estimated value of the mean blood pressure. Having found the average cycle length T^*^ by minimising $f(T)$, we then choose our delay parameter to be either $\tau=T^*/3$ or $\tau=2T^*/3$.

In practice, we work with uniformly sampled data in which case *T* has to be chosen as a multiple of the timestep and the norm is a vector norm of the data in a time window. We also restrict the range of possible *T* values based on a known physiological range of cycle length. For a healthy mouse, the heart rate is typically in the range of 450–750 bpm (Starr *et al* 2014) so we choose a slightly larger range of 400–800 bpm which corresponds to a cycle length in the range 75–150 ms.

**Figure 6.**
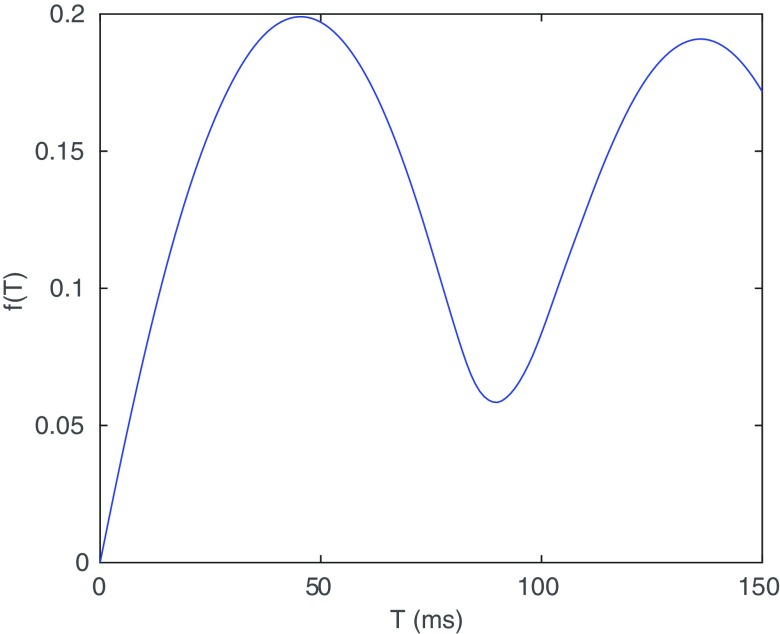
Plot of the function $f(T)$ given by (13) for the data in figure 2 (top).

For the data shown in figure 2 (top), a plot of $f(T)$ is shown in figure 6. This has a minimum of $0.058399$ at *T*  =  T^*^  =  90 ms and so this is the average cycle length that we use. Taking $\tau=T^*/3=30$ ms, we then obtain the plot in the $(v, w)$ plane shown in figure 2 (bottom left). We note that a cycle length of 90 ms corresponds to 111 cycles in a 10 s window. The number of peaks in the data in figure 2 (top) is 110 and so we have got an accurate estimate of the average cycle length. The autocorrelation function also has a peak at *T*  =  90 ms, and so the same value is obtained using both methods in this case. We are therefore able to accurately extract the average cycle length (and hence the average heart rate) from a noisy and non-stationary signal.

### 2.5. Construction of the density

A long trajectory plotted in the $(v, w)$ plane very quickly gives a messy result and so for the third step of our method, we turn the trajectory in the $(v, w)$ plane into a density since this shows the regions that the trajectory often returns to and other regions that are only visited infrequently. This provides information about the variability of the waveform shape in each cycle, which may relate to how the cardiovascular system is changing or adapting to pathophysiological or physiological changes. It can also indicate the speed of motion at various regions of the attractor, since greater speed corresponds to a lower density and vice versa.

We generate the density on a square grid of boxes in the plane and normalise the volume to be one. Some nice examples of density plots for the Lorenz attractor in three-dimensions are given in Bürkle *et al* (1999). The density corresponding to the attractor in figure 2 (bottom left) is shown in figure 2 (bottom right). In this case, a grid of $100\times 100$ boxes was used to generate the density.

### 2.6. Generation of the time traces

The final step of our approach is to extract key features of the density and trace them out as a time window is moved through the data. In some cases, the features of the density may relate directly to features of the signal (see section 4 for some examples), but this is not a necessary condition and we can also extract features of the density that do not have any obvious association with any particular aspect of the signal.

Initially, there are three obvious features of the density that we can always determine.

1. The first step in this approach is always to find the average cycle length for each window of data, and so this is the first measure that we extract. For blood pressure data, the cycle length is the time between heart beats. A related and more commonly used quantity is the heart rate and so we could plot this quantity rather than the average cycle length.
2. We find the average cycle length by minimising $f(T)$ given by (13). The second obvious quantity to follow is $f(T_{\rm min})$, where *T*_min_ is the average cycle length that minimises *f*. This quantity gets closer to zero as the data become closer to periodic, so this can be regarded as a measure of how close the data in the time window are to being periodic.
3. From the density, the simplest quantity that can be extracted is the maximum value and so this measure can be traced with the moving window. The maximum value may depend on a few features of the density. Since the volume of the density is normalised to one, if the attractor gets larger, then the maximum is likely to decrease. Similarly, if the trajectory is close to periodic, then the attractor will be quite narrow and so will have a higher density.

An example of some blood pressure data together with a plot of these three traces is shown in section 5 (see figures 12 and 14).

### 2.7. Diagnosis

The aim of this approach is to be able to diagnose various conditions by monitoring the blood pressure data, and to describe pathological changes in the signal over time. The final step in this process, having extracted various measures over time from the density, is to determine the aspects of these measures that are associated with particular diseases. This process will involve classification methods based on machine learning. See Lyle *et al* (2017) for a simple example of this classification process. By generating traces for multiple measures derived from the density, we anticipate that there will be a unique ‘signature’ in the derived traces for a variety of physiological conditions which will allow early detection of the underlying changes in the cardiovascular system control mechanisms. We anticipate that our approach will detect changes earlier than the macrophysiological changes observed using conventional analysis, which can commonly be subject to over or underinterpretation due to baseline variation, averaging and data exclusion.

## 3. Idealised blood pressure signal

We would like to relate particular properties of the attractor with features of the blood pressure signal in order to give physiological interpretation to some of the measures that we extract from the attractor. To help understand this relationship, we consider an idealised blood pressure signal that is piecewise polynomial and periodic. We do of course recognise that a blood pressure signal is not exactly periodic, but studying an idealised periodic signal can help provide insight into various properties of the attractor.

In this section, we assume that $x(t)$ is continuous and periodic with period 1. As noted in section 2.4, the trajectory in the three-dimensional phase space and its projection onto the two-dimensional $(v, w)$ plane corresponding to a periodic signal are both closed orbits. We will also restrict attention to the two optimal values of *τ*, namely $\tau=1/3$ and $\tau=2/3$ (see section 2.4).

### 3.1. Piecewise linear signal

**Figure 7.**
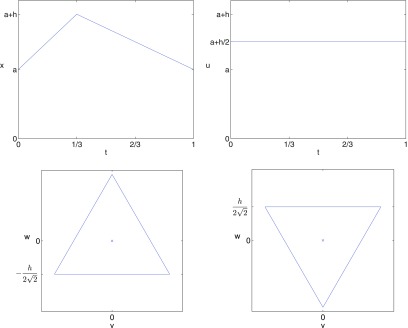
Top left: A piecewise linear periodic signal. Top right: Plot of $u(t)$ for $\tau=1/3, 2/3$. Bottom left: Plot of *v* against *w* for $\tau=1/3$. Bottom right: Plot of *v* against *w* for $\tau=2/3$.

We first consider the simple case where the signal $x(t)$ is piecewise linear. The blood pressure increases during systole and decreases during diastole. The ratio of these two phases varies with heart rate. For the human heart, systole typically lasts for approximately 34% of the cycle at a heart rate of 75 beats/minute and this increases to 53% of the cycle when the heart rate increases to 200 beats/minute (Barrett *et al* 2010, table 31-1, p 512). For comparison with the ratio for the slower heart rate, we consider the idealised case where the signal consists of two linear segments with the break between them occurring at *t*  =  1/3, as shown in figure 7 (top left). The function $x(t)$ in this case is given by

14 \begin{align*} \newcommand{\e}{{\bf e}} \displaystyle \label{pl1} x(t)=\left\{\begin{array}{@{}ll@{}} a+3ht, &amp; 0\leqslant t\leqslant \frac{1}{3} \nonumber \\[3mm] a+\frac{3h}{2}(1-t), &amp; \frac{1}{3}\leqslant t\leqslant 1. \end{array}\right. \nonumber \end{align*}

For this function, we have the following result.

Lemma 3.1. Let $x(t)$ be the piecewise linear function defined in (14) and shown in figure 7 (top left).

(i) If $\tau=1/3$ then the trajectory in the $(v, w)$ plane, as shown in figure 7 (bottom left), is an equilateral triangle centred on the origin with one edge given by
  \begin{align*} \newcommand{\e}{{\bf e}} \displaystyle w=-\frac{h}{2\sqrt{2}},~~v\in\left[-\frac{\sqrt{3}h}{2\sqrt{2}},\frac{\sqrt{3}h}{2\sqrt{2}}\right]. \nonumber \end{align*}
  The trajectory cycles in a clockwise direction with uniform speed. Moreover, $u(t)=a+h/2$, which is the midpoint of the range of the cycle (see figure 7 (top right)).
(ii) If $\tau=2/3$ then the trajectory in the $(v, w)$ plane, as shown in figure 7 (bottom right), is an equilateral triangle centred on the origin with one edge given by
  \begin{align*} \newcommand{\e}{{\bf e}} \displaystyle w=\frac{h}{2\sqrt{2}},~~v\in\left[-\frac{\sqrt{3}h}{2\sqrt{2}},\frac{\sqrt{3}h}{2\sqrt{2}}\right]. \nonumber \end{align*}
  This triangle is the reflection in the *v* axis of the one for $\tau=1/3$. The trajectory cycles in an anticlockwise direction with uniform speed. Again, $u(t)=a+h/2$, which is the midpoint of the range of the cycle (see figure 7 (top right)).

We note in this case, as with the sinusoidal orbit considered in section 2.4, that the size of the orbit in the $(v, w)$ plane is proportional to the amplitude of the signal.

We proved in theorem 2.3 that the orbit in the $(v, w)$ plane will have ${\bf Z}_3$ symmetry if the signal is periodic and $\tau=1/3$. However, when *x* is the piecewise linear signal that we have just considered, the orbit has ${\bf D}_3$ symmetry, not just ${\bf Z}_3$ symmetry, since it is an equilateral triangle. The conditions that must hold more generally on *x* for the extra reflectional symmetry to be present can be derived, but they are not particularly enlightening and so we do not include them here.

### 3.2. Piecewise quadratic signal

When processing mouse blood pressure data, we have observed two key differences in the attractor from the equilateral triangle described above. In some cases, a triangular attractor is rotated clockwise in the $(v, w)$ plane by a small amount. Also, the motion around the attractor is often not uniform, since the density along the edges is not constant. Along the bottom edge of the attractor, it is frequently observed that the density is higher at the left than at the right. This suggests that the motion along the bottom edge (from right to left) is initially fast and gradually slows down. The other two edges show a similar pattern. We therefore now consider what changes to the idealised piecewise linear signal have to be made to introduce these effects.

In the discussion below, the shape of the waveform in terms of its convexity or concavity is central. However, there are two conflicting definitions of these terms, so we will now clarify the definition that we will use. In mathematics, a convex function is convex downward, which means that for any point *z* between two points *x* and *y*, the point $(x, f(z))$ lies below the straight line joining the points $(x, f(x))$ and $(y, f(y))$. A simple example of a convex function is *f*(*x*)  =  *x*^2^. However, the common definition of convexity refers to something that curves outwards, such as a convex lens that bulges in the middle. Consider the simple example of the downstroke of a blood pressure signal being a decaying exponential function. In this case, the downstroke is a convex function. However, in the context of the blood pressure signal, it appears visually to be curving inwards, and hence could be described as concave. We will fix on the latter, more intuitive, definition rather than the strict mathematical definition, and so will describe such a curve as concave.

#### 3.2.1. A rotated triangle

We first consider the type of signal that gives a rotated equilateral triangle as the orbit in the $(v, w)$ plane with uniform motion.

Lemma 3.2. Let $\tau=1/3$ and $\beta\in{\bf R}$. We define the piecewise quadratic signal

**Figure 8.**
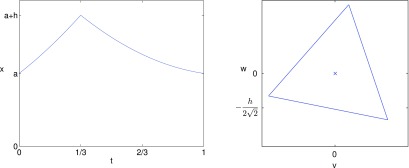
A piecewise quadratic signal with $\beta=0.2$ that gives a rotated equilateral triangle in the $(v, w)$ plane with uniform motion. Left: The piecewise quadratic signal $x(t)$. Right: A plot of the trajectory in the $(v, w)$ plane with $\tau=1/3$.

15 \begin{align*} \newcommand{\e}{{\bf e}} \displaystyle \label{pqfn} x(t)=\left\{\begin{array}{@{}ll@{}} a+\frac{3ht}{2}\left(2+\sqrt{3}\beta(3t-1)\right), &amp; 0\leqslant t\leqslant\frac{1}{3} \nonumber \\[3mm] a+\frac{3h}{2}(1-t)\left(1-\sqrt{3}\beta(3t-1)\right), &amp; \frac{1}{3}\leqslant t\leqslant 1 \end{array}\right. \nonumber \end{align*}

which is shown in figure 8 (left). In the $(v, w)$ plane, the orbit is a rotated equilateral triangle which is traversed with uniform speed with the bottom edge given by

\begin{align*} \newcommand{\e}{{\bf e}} \displaystyle w=-\beta v-\frac{h}{2\sqrt{2}}(1+\beta ^2),~~v\in\left[-\frac{(\sqrt{3}+\beta)h}{2\sqrt{2}},\frac{(\sqrt{3}-\beta)h}{2\sqrt{2}}\right]\nonumber \end{align*}

as shown in figure 8 (right). The length of the sides of the triangle is

\begin{align*} \newcommand{\e}{{\bf e}} \displaystyle \frac{\sqrt{3}h}{\sqrt{2}}\sqrt{1+\beta ^2}.\nonumber \end{align*}

If the bottom edge of the triangle is rotated by *θ* from the horizontal in a clockwise direction, then $\beta =\tan\theta$ and so $\sqrt{1+\beta ^2}=\sec\theta$ provided that $0\leqslant\theta&lt;\pi/2$.

The quadratic function $x(t)$ on the interval $t\in[0, 1/3]$ is monotonically increasing provided that $\vert \beta\vert &lt;2/\sqrt{3}$ (or equivalently, $\vert \theta\vert &lt;0.8571~{\rm rad}, 49.11^\circ$) and the quadratic function $x(t)$ on the interval $t\in[1/3, 1]$ is monotonically decreasing provided that $\vert \beta\vert &lt;1/(2\sqrt{3})$ (or equivalently, $\vert \theta\vert &lt;0.2810~{\rm rad}, 16.10^\circ$).

Clearly, setting $\beta=0$ in lemma 3.2, we revert to the piecewise linear function which was considered in lemma 3.1 which results in an equilateral triangle with horizontal base in the $(v, w)$ plane.

The piecewise quadratic function defined by (15) solves the problem of finding the signal corresponding to a rotated attractor with uniform motion. However, it is composed of two concave functions when $\beta&gt;0$ (see figure 8). It is more common in the blood pressure signal to see a convex function as the blood pressure rises, followed by a concave function as it falls. Thus, we now consider the general case of two piecewise quadratic functions with one convex and the other concave. In particular, we define the piecewise quadratic signal

16 \begin{align*} \newcommand{\e}{{\bf e}} \displaystyle \label{pqfn2} x(t)=\left\{\begin{array}{@{}ll@{}} x_1(t), &amp; 0\leqslant t\leqslant\frac{1}{3} \nonumber \\ x_2(t), &amp; \frac{1}{3}\leqslant t\leqslant 1 \end{array}\right. \nonumber \end{align*}

where

\begin{align*} \newcommand{\e}{{\bf e}} \displaystyle x_1(t)=a_1t^2+b_1t+c_1,~~x_2(t)=a_2t^2+b_2t+c_2.\nonumber \end{align*}

We impose the consistency relations $x_1(0)=x_2(1)=a$ and $x_1(1/3)=x_2(1/3)=a+h$ which can be solved for *b*_1_, *c*_1_, *b*_2_ and *c*_2_ giving

17 \begin{align*} \newcommand{\e}{{\bf e}} \displaystyle \label{pq2constr} b_1=-\frac{1}{3}a_1+3h,~~c_1=a,~~b_2=-\frac{4}{3}a_2-\frac{3}{2}h,~~c_2=\frac{1}{3}a_2+a+\frac{3}{2}h. \nonumber \end{align*}

Clearly this leaves *a*_1_ and *a*_2_ as free parameters.

Lemma 3.3. Consider the periodic, piecewise quadratic signal given by (16) with constraints given by (17) and let $\tau=1/3$. Then

\begin{align*} \newcommand{\e}{{\bf e}} \displaystyle v=Aw^2+Bw+C~{\rm for}~\frac{2}{3}\leqslant t\leqslant 1\nonumber \end{align*}

where

\begin{align*} \newcommand{\e}{{\bf e}} \displaystyle A=\frac{3\sqrt{3}}{\sqrt{2}}\left(\frac{a_2-a_1}{a_2^2}\right).\nonumber \end{align*}

If *x*_1_ is convex (*a*_1_  <  0) or linear (*a*_1_  =  0) and *x*_2_ is concave (*a*_2_  >  0), then *A*  >  0 and so this edge bows outwards. Also, $w(1)-w(2/3)=\sqrt{2}a_2/9$ and so if *x*_2_ is concave (*a*_2_  >  0), then the straight line joining the two ends of this curve in the $(v, w)$ plane has negative slope which is given by $-2a_2/(9\sqrt{3}h)$.

The two remaining sides of the closed orbit can be obtained by rotation of this quadratic function by $2\pi/3$ and $4\pi/3$ about the origin.

We note from this result that the clockwise rotation of the corner points of the attractor is due only to the second quadratic function *x*_2_ being concave. Conversely, any signal for which the decline is convex would of course give rise to an anticlockwise rotation of the attractor. Also, if $a_2&lt;a_1$, then the edges of the orbit will bow inwards rather than outwards.

**Figure 9.**
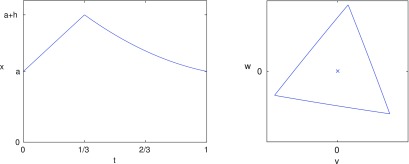
A periodic signal with linear upstroke (*a*_1_  =  0) and quadratic concave downstroke (*a*_2_  >  0) that gives a rotated (slightly curved) triangle in the $(v, w)$ plane. Left: The piecewise signal $x(t)$. Right: A plot of the orbit in the $(v, w)$ plane.

An example with linear upstroke but quadratic downstroke is shown in figure 9. The curve on the edges of the triangle is hardly visible. Theoretically, the motion along each edge of the triangle is not uniform, but for this example, it is very close to being uniform. Thus, a linear upstroke with a quadratic downstroke gives a rotated triangle with edges that are almost straight and which has almost uniform motion.

This example assumes a very specific form of the signal. More generally, assuming that the downstroke is approximately two thirds of the cycle length, the corner point of the attractor with *v*  >  0, *w*  <  0 occurs when the point *y* is at the peak of the signal and *x* is halfway down the downstroke. The corresponding value of *w* (see (2)) is related to the difference in height between these two points. The next corner, with *v*,*w*  <  0, occurs when *x* is at the minimum and *y* is halfway along the downstroke and again the value of *w* is related to the difference in height of these two points. Moving from the first corner to the second, the magnitude of *w* will decrease, resulting in a clockwise rotation of the corner points, provided that the difference in height between the midpoint of the downstroke and the minimum point is smaller than the difference of the maximum point and the midpoint of the downstroke. This occurs provided that the midpoint is below a straight line joining the maximum and minimum points. Clearly this holds if the downstroke is concave, as discussed above, but will also hold in many other cases where the downstroke is more variable.

Biologically, a concave downstroke, corresponding to the clockwise rotation of the triangle in figure 9, has been observed in mammals and may arise from peripheral wave reflections. Quantification of dynamic changes may therefore provide information about changes in the resistance or compliance of the vascular network which in turn may provide additional information which may predict patient decompensation or differential responses to drug treatment (London and Pannier 2010, Thiele and Durieux 2011, Alastruey *et al* 2014).

#### 3.2.2. Non-uniform motion

We now consider what changes have to be made to the piecewise linear signal in order to generate an orbit that is an equilateral triangle with a horizontal base but with non-uniform motion along the edges.

We can understand this case from the previous example by taking the signal given in (16) (with the constraints in (17)) but setting *a*_2_  =  0 so that the downstroke is a linear function. In this case, it is easily verified that along the bottom edge, $w(t)=-h/\left(2\sqrt{2}\right)$ is constant and so the orbit in the $(v, w)$ plane is again an equilateral triangle with horizontal bottom edge. However, in this case, the function *v* is given by

\begin{align*} \newcommand{\e}{{\bf e}} \displaystyle v(t)=-\sqrt{\frac{2}{3}}a_1t^2+\frac{1}{3\sqrt{6}}(10a_1-27h)t+\frac{1}{6\sqrt{6}}(45h-8a_1),~~t\in[2/3,1]. \nonumber \end{align*}

Differentiating gives

\begin{align*} \newcommand{\e}{{\bf e}} \displaystyle v'(t)=-\frac{2\sqrt{2}}{\sqrt{3}}a_1t+\frac{1}{3\sqrt{6}}(10a_1-27h). \nonumber \end{align*}

Now $v'(t)&lt;0$ for all $t\in[2/3, 1]$ since the orbit moves from right to left along the bottom edge. Thus, the velocity along this edge will go from fast to slow if $v'(t)$ has positive slope and this occurs provided that *a*_1_  <  0.

**Figure 10.**
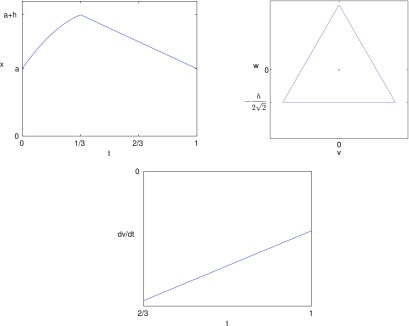
A periodic signal with quadratic convex upstroke (*a*_1_  <  0) and linear downstroke (*a*_2_  =  0) that gives an equilateral triangle with horizontal base in the $(v, w)$ plane but with non-uniform motion. Top left: The piecewise signal $x(t)$. Top right: A plot of the trajectory in the $(v, w)$ plane. Bottom: A plot of ${\rm d}v/{\rm d}t$ shows that the velocity along the edge gradually slows.

Thus, we conclude that if the upstroke is quadratic and convex (*a*_1_  <  0) and the downstroke is linear (*a*_2_  =  0), then the orbit is an equilateral triangle with horizontal bottom edge but with non-uniform motion where, along each edge, the motion is initially fast but gradually slows down. This is illustrated in figure 10.

These last two examples show that a clockwise rotation of the attractor is associated with the downstroke of the idealised signal becoming concave, while a triangular attractor without any rotation but with non-uniform motion, which can be observed from changes in the density along the edge, is associated with the upstroke becoming convex. Typical blood pressure signals contain both these features.

These examples show how particular features of the signal are reflected in properties of the attractor in the $(v, w)$ plane and provide candidates for measures derived from the attractor for monitoring specific features of the signal.

## 4. Attractor measures

The relationship between the waveform morphology and the resultant attractor means that numerous scalar measures that describe the attractor features must by definition relate to aspects of the waveform shape. It is likely that different pathologies and physiological states will generate a signature of corresponding attractor features and we anticipate that this would enable identification and discrimination between different pathologies.

In the previous section, we related various features of a periodic, idealised signal to properties of the reconstructed attractor. We now use these results as the basis for deriving further measures of the attractor density generated by blood pressure data that can be monitored with a moving time window. Note that in practice, we often refer to the ‘attractor density’ as simply the ‘attractor’.

### 4.1. Pulse pressure measures

For the piecewise linear signal defined by (14), we showed in lemma 3.1(i) that the trajectory in the $(v, w)$ plane is an equilateral triangle with horizontal bottom edge at $w=-h/\left(2\sqrt{2}\right)$. The size of the triangle is therefore determined by the pulse pressure (amplitude) of the signal. Thus, for an attractor generated by a blood pressure signal, we can monitor the size of the triangle and derive from this a measure of the pulse pressure. The thickness of the triangular band also varies and so measures related to this thickness will provide information regarding variability in the shape of the waveform.

To derive measures related to this band thickness, we want to include contributions from all three sides of the attractor. To achieve this, if $D(\tau)$ is the density on a square grid with delay parameter *τ*, then we define

**Figure 11.**
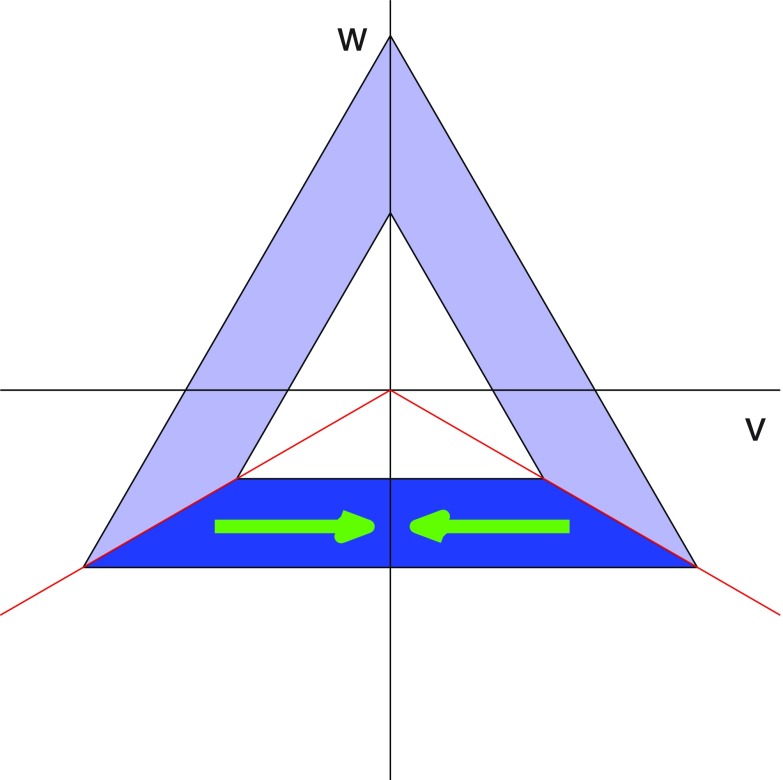
The dark blue region of the attractor $D_s(\tau_1)$ is summed in the *v* direction (green arrows) to give a density in *w*.

\begin{align*} \newcommand{\e}{{\bf e}} \displaystyle D_s(\tau)=\frac{1}{3}(D_1(\tau)+D_2(\tau)+D_3(\tau))\nonumber \end{align*}

where $D_1(\tau)=D(\tau)$ and $D_2(\tau)$ and $D_3(\tau)$ are density matrices derived from the data rotated in the $(v, w)$ plane by $2\pi/3$ and $4\pi/3$ respectively. (We note that it is more accurate to generate a new density matrix from rotated data than to perform a rotation of the density matrix since rotation of a square grid by $2\pi/3$ does not readily map onto the original grid.) The matrix $D_s(\tau)$ is the density (on a square grid) of an attractor that has ${\bf Z}_3$ symmetry by construction. Clearly, one edge of $D_s(\tau)$ contains information from all three edges of $D(\tau)$. Thus, it is sufficient to consider one third of it, as shown by the dark blue region in figure 11, since this contains an average of the three sides of the original density $D(\tau)$. We take this section and sum the entries in the density matrix along the *v* direction to give a new density function $\tilde d(w)$ which depends only on (negative values of) *w*. Since the magnitude of *w* along this edge is related to the pulse pressure (amplitude) *h*, then we define $h=-2\sqrt{2}w$ and express the density in terms of this quantity as $d(h)=\tilde d(-h/(2\sqrt{2}))$. This gives a density function which shows the distribution of the pulse pressures in the signal. From the density function $d(h)$ we can derive a number of scalar measures such as

1. the first, second (median) and third quartiles;
2. the maximum and minimum of *h* where the density is non-zero;
3. a threshold maximum and minimum, which are the maximum and minimum values of *h* such that the density is greater than a specified threshold value.

This combination of values gives a good indication of the shape and spread of the density $d(h)$. We note that if the data contain occasional irregular events, then this would appear in the attractor as brief excursions away from the triangle and so would result in a large difference between the maximum/minimum and the threshold maximum/minimum. Thus, a large difference in these quantities is an indicator of rare irregular events occurring in the window of data. This difference will persist as long as the rare event is in the time window, and so will occur in the time traces for a time approximately the same as the window length.

A further measure that we derive is the maximum value of the density $d(h)$. While a narrow density will give rise to a high maximum value, when the density is wider, there may or may not be a large peak in the density, and so this measure does not directly correlate with the width measures described above.

Physiologically, changes in pulse pressure are known to correlate with a number of cardiovascular disorders, including septic shock. Accurate quantification of this parameter is therefore important for clinical diagnosis. Whether the extraction of pulse pressure using this novel method is superior to currently used methods from noisy, non-stationary data remains to be determined.

### 4.2. Rotation

As discussed in section 3.2.1, the triangular attractor is sometimes rotated in a clockwise direction by a small amount. We showed in lemma 3.3 that this arises due to curvature in the downstroke of the waveform. Another measure that we consider therefore is the angle of rotation of the triangle since this provides information regarding this aspect of the waveform. We consider two methods for finding this angle.

In the first method, for a given angle *θ*, we rotate the data in the $(v, w)$ plane in an anticlockwise direction by *θ* and find the density function $d(h)$ described above. We then define a function $f(\theta)$ as the width of the band which is found as the difference between the maximum and minimum values of *h* that have a non-zero value for the density. The angle of rotation of the attractor is then found by minimising $f(\theta)$. This method is equivalent to drawing the largest possible equilateral triangle that fits inside the attractor together with the smallest possible equilateral triangle that fits outside the attractor, both rotated clockwise by *θ*, and then finding the width of the gap between the two triangles. This width will generally be a minimum when the correct angle of rotation of the attractor is used. However, one drawback with this method is that it is influenced by rare irregular events that push out the maximum and minimum boundaries of the attractor and, in some cases, this can lead to the wrong value of *θ* being obtained. An alternative would be to use the threshold maximum and minimum boundaries instead.

An alternative method is to again rotate the data in an anticlockwise direction by *θ* and construct the density function $d(h)$ as above. We then define the function $g(\theta)=\max\nolimits_h d(h)$ and maximise this function to find the optimal value of *θ*. This method works since the density function $d(h)$ will be at its narrowest, and hence have the greatest maximum, when the bottom edge of the rotated triangle is horizontal. If the wrong angle is used, then the bottom edge of the triangle will not be horizontal and so summing the density in the *v* direction will result in a more widely spread density function. The advantage of this method is that the optimal angle is determined by the dominant behaviour in the signal, not by the rare events as in the first method. Thus, we use this method to determine the angle of rotation of the attractor.

Having found the angle of rotation *θ*, we then note that if the triangular orbit shown in figure 8 (bottom) is rotated in an anticlockwise direction by *θ* then, from the results in lemma 3.2, we find that the base of the triangle is defined by $w=-h\sec\theta/(2\sqrt2)$. Thus, for the triangular attractor derived from data, we find the value of *θ* and then derive the pulse pressure distribution by defining $d(h)=\tilde d(-h\sec\theta/(2\sqrt{2}))$, which is well-defined provided that $\theta\in[0, \pi/2)$.

## 5. Analysis of blood pressure data

**Figure 12.**
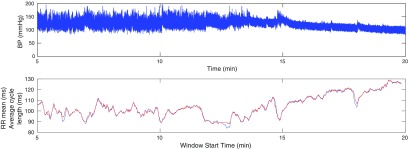
Top: 15 s of blood pressure data. Bottom: The mean of the RR intervals extracted from the BP data (blue) and the average cycle length found by minimising $f(T)$ (red) using a moving 10 s window.

As a practical example of the method described above, we have applied it to blood pressure data sampled at 1000 Hz that have been collected from a healthy, conscious mouse using an implanted radiotelemetry device coupled to the Data Sciences International A.R.T. acquisition system (DSI Dataquest ART System 2015, Starr *et al* 2014). This current gold standard technique allows high quality physiological data with very little artefactual noise to be collected remotely from unrestrained animals left undisturbed in their home cages, minimising the confounding effects of stress and thereby maximising the physiological relevance of the data (Nandi *et al* 2012, Starr *et al* 2014). These data show cyclic behaviour due to the regular heartbeat but the signal is certainly not periodic due to the many other physiological factors that result in variation in this signal over time (see figures 1 and 12). The beat-to-beat interval is approximately 100 ms, corresponding to a heart rate of 600 beats per minute, and so there are around 100 data points per cycle. The sample rate of 1000 Hz is high for these blood pressure data (but may be required to accurately capture the R-wave in an ECG signal). Our approach works equally well with a lower sampling frequency for these data. More generally the important criterion is to have sufficient data points per cycle to accurately represent all the features of the waveform. For these blood pressure data, a sampling frequency of 500 Hz or 250 Hz would result in approximately 50 or 25 points per cycle respectively, which should still be sufficient to define the waveform and hence generate a clear attractor. Clearly, fewer data points per cycle will result in a less precise attractor, which could affect some properties such as the maximum density. However, other large scale features, such as the rotation, should still be clearly discernible.

The only preprocessing that we perform on the data is the simple removal of any obvious outliers that are outside a specified range. These typically arise from electrical interference which results in spikes in the data. This is in contrast to the preparation of data for HRV analysis in which the heart beats have to be identified in the data in order to find the beat-to-beat (RR) intervals. These interval lengths are then further refined, for example by excluding an interval that differs from the previous one by more than 20% (Cam *et al* 1996), to give the normal beat-to-beat (NN) intervals. The 1996 HRV task force review (Cam *et al* 1996) recommended that ‘manual editing of the RR data should be performed to a very high standard’, and that automatic filtering of the RR intervals to give the NN intervals ‘should not replace manual editing’ as it can ‘have undesirable effects leading potentially to errors’. However, the large quantity of data that is now collected means that manual detection and filtering becomes impractical. Hence it is essential to have computational approaches that will not only handle these large data sets but that will also minimise human error and/or bias introduction.

We have applied our method to 15 min of blood pressure data from a healthy mouse which is shown in figure 12. A moving window of length 10 s, which contains approximately 100 cycles, is used. The first step is to determine the average cycle length in the window of data, as discussed in section 2.4. For this example, we find the mean of the RR intervals (peak to peak distances) and we also find the average cycle length using our approach of minimising the function $f(T)$ given by (13). Both of these are shown in figure 12 and it can be seen that they are in good agreement. The advantage of minimising $f(T)$ is that it is not necessary to identify individual points on the signal. The time delay parameter *τ* for each window is taken to be one third of the average cycle length. Clearly the average heart rate can be derived from this average cycle length. We note that the average cycle length fluctuates around 100 ms for the first 12 min and then gradually increases to around 125 ms over the last 8 min interval, which clearly corresponds to a gradually decreasing heart rate.

**Figure 13.**
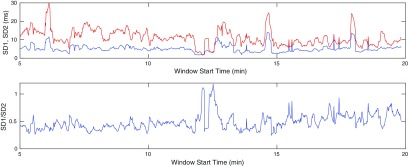
HRV measures derived from interval data in a moving 10 s window. Top: SD1 (blue) and SD2 (red). Bottom: ${\rm SD}12={\rm SD}1/{\rm SD}2$.

Our aim is to compare some of the many HRV measures with our attractor reconstruction (AR) measures. For the HRV measures, we have chosen three quantities that are derived from a Poincaré plot of successive pairs of interval lengths, namely SD1, SD2 and the ratio ${\rm SD}12={\rm SD}1/{\rm SD}2$. It has been shown that SD1 is a scaled version of SDSD, the standard deviation of successive differences of the interval lengths, and that ${\rm SD2}^2=2{\rm SDRR}^2-{\rm SD1}^2$, where SDRR is the standard deviation of the interval lengths (Brennan *et al* 2001). It is also known that SD1 is related to short term interval variation while SD2 is related to long term interval variation. Thus, the ratio SD12 gives the ratio of short term to long term variability (Acharya *et al* 2006). The peak-to-peak interval lengths were extracted for each window of data and these three quantities evaluated. The plot as the time window moves through the data is shown in figure 13. For this example, SD1 is smaller than SDRR and so ${\rm SD2}\simeq\sqrt{2}{\rm SDRR}$.

**Figure 14.**
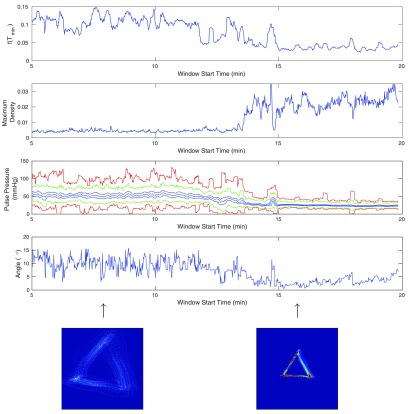
Attractor reconstruction measures derived using all of the BP data shown in figure 12 with a moving 10 s window. Top: The function $f(T)$ defined by (13) with $\bar x=100$ evaluated at the average cycle length *T*_min_. Second: The maximum density. Third: Pulse pressure measures (red: maximum, minimum; green: maximum and minimum above the threshold; blue: first, second (median) and third quartiles). Fourth: Angle of the attractor. Bottom: The attractors at time *t*  =  8 min and *t*  =  16 min.

**Figure 15.**
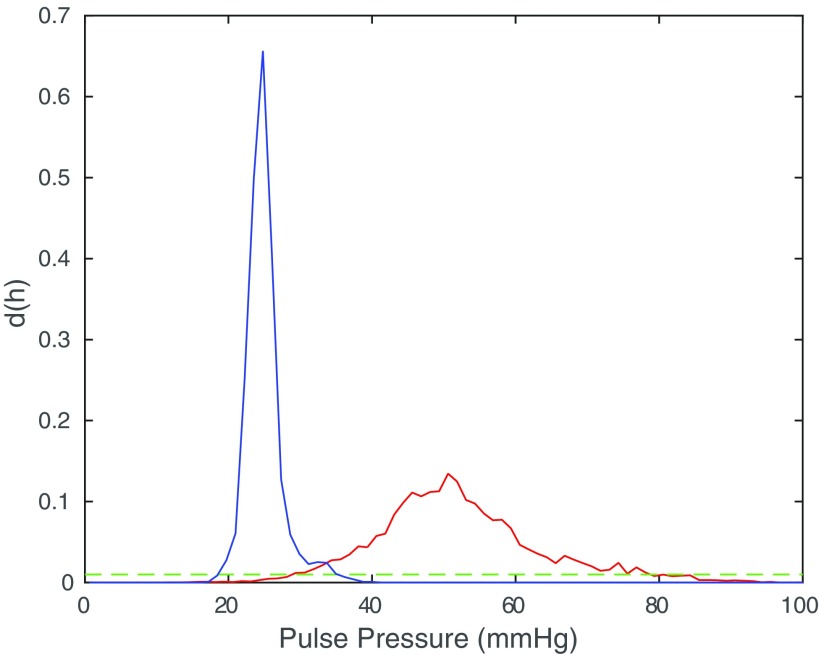
The densities $d(h)$ for the two attractors shown in figure 14 at times *t*  =  8 min (red) and *t*  =  16 min (blue). The threshold is shown in green.

For the attractor reconstruction (AR) measures, we find the measure of periodicity $f(T_{\rm min})$ (see (13)), the maximum density of the attractor, the pulse pressure measures and the angle of the attractor. These are all shown in figure 14. Two attractors at times *t*  =  8 min and *t*  =  16 min are also shown in figure 14 and it is obvious visually that these are very different in many respects. The attractor at *t*  =  8 min is much larger than the one at *t*  =  16 min and the sides of the attractor at *t*  =  8 min are quite broad, compared with the narrow sides at *t*  =  16 min. Since the total density is normalised to one, both these effects result in the maximum density at *t*  =  8 min being much lower than the maximum density at *t*  =  16 min. The densities $d(h)$ for the two attractors are shown in figure 15, and the pulse pressure measures are derived from these densities. The sides of the *t*  =  8 min attractor are much broader than the sides of the *t*  =  16 min attractor, and this is reflected in the different width of the two densities. We also note that the angle of rotation for the *t*  =  8 min attractor is greater than that for the *t*  =  16 min attractor.

For these data, we note that the HRV measures are noisy but show little change over the 15 min interval. There is a slight reduction in SD2 over the last 5 min, which gives a corresponding slight increase in the ratio SD12, but these are very small variations. So while there is a gradual reduction in heart rate, there is very little change in its variability.

In contrast, all of the AR measures show a sharp and significant change at time around 13.5 min. The periodicity measure decreases significantly indicating that the data change from quite variable to being much more periodic. This change is evident from the two attractors that are shown at the bottom of figure 14. The attractor at time *t*  =  8 min is a thick band indicating that the data contain a lot of variability, while the attractor at time *t*  =  16 min is much thinner, which shows that the data are much closer to being periodic. This change also explains the sudden jump in the maximum density as the thin attractor has a much more concentrated density than the earlier thicker attractor. The pulse pressure measures, derived from the thickness of the sides of the attractor, also show a dramatic change at the same time point in two regards. Firstly, there is a reduction in the median pulse pressure from around 50 mmHg to about 25 mmHg, and secondly, the variability is drastically reduced as can be seen from the reduction in the spread of the various lines plotted.

While the changes in the above measures are all somewhat related to the same reduction in variability in the data, the angle property of the attractor is not related to this change in variability, but is associated with a change in the concavity of the downstroke. This angle also shows a significant change at around the same time point, and the reduction in angle can also be seen in the two attractors in figure 14.

In summary, when looking at the condensed raw blood pressure signal, it can be seen clearly by eye that pulse pressure changes, as does the variability of the waveform shape. The heart rate measure also shows a gradual decrease. In the clinical setting however, data would not be viewed in this way and thus the subtleties of this variation could be missed. Interestingly, the standard HRV measures are unable to detect robust changes, suggesting that beat to beat variability may be preserved. In contrast, our attractor reconstruction method demonstrates that the ‘waveform shape variability’ is altered significantly and rapidly as quantified by all of our derived measures, and so we are able to detect quantifiable measures of change above and beyond traditional approaches.

## 6. Artificial signal with variability

As a final example, we return to an artificial, piecewise linear signal, similar to the one which we considered in section 3.1. We again have a signal that consists of a linear upstroke and a linear downstroke with fixed peak-to-peak (and trough-to-trough) intervals. The time interval for the upstroke will again be half that of the downstroke. However, we now consider two different scenarios:

1. The slope of the downstroke is fixed, while the slope of the upstroke is varied randomly.
2. The slope of the upstroke is fixed, while the slope of the downstroke is varied randomly.

**Figure 16.**
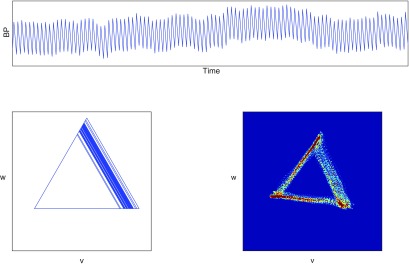
Top: Piecewise linear signal with randomly varying slope of the upstroke and fixed slope of the downstroke. Bottom left: The corresponding attractor in the $(v, w)$ plane. Bottom right: A similar attractor derived from the data shown in figure 12 (top) at time *t*  =  19.68 min.

**Figure 17.**
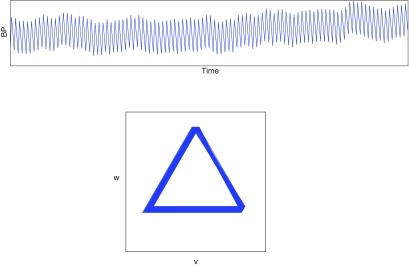
Top: Piecewise linear signal with fixed slope of the upstroke and randomly varying slope of the downstroke. Bottom: The corresponding attractor in the $(v, w)$ plane.

Thus, in the first case, there is variability only in the upstroke whereas in the second case, there is variability only in the downstroke. Plots of the artificial data and the corresponding trajectories in the $(v, w)$ plane are shown in figures 16 and 17.

For this example, we have a fixed cycle length and so a fixed heart rate which implies that there is no heart rate variability at all. However, the two signals that we consider have quite distinct characteristics and these are reflected in the different types of attractor that are generated. We note that the attractor at *t*  =  8 min in figure 14 is quite similar to the attractor in figure 17 and we have included a density in figure 16 (which comes from the data shown in figure 12 at *t*  =  19.68 min) which is similar to the attractor generated by the artificial data.

The ability to model changes using a piecewise linear signal and then compare them to physiological data proves to be a powerful approach. Indeed, the changes observed in figure 16 are observed in healthy human data and we believe that they relate to variability in cardiac contractility in the healthy state.

Clearly, there are many more ways that an artificial piecewise linear signal could be generated, but exploring more possibilities is beyond the scope of this work.

## 7. Application to other signals

**Figure 18.**
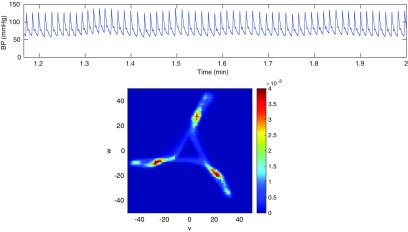
Top: A sample of human blood pressure data. Bottom: The corresponding attractor in the $(v, w)$ plane.

The attractor reconstruction analysis of blood pressure waveforms that we have described is a robust method that can be applied to any continuous, approximately periodic waveform. A sample of human blood pressure data, obtained by FINApress fingertip plethysmography (Kasprowicz *et al* 2010), together with the corresponding attractor is shown in figure 18. It can also be applied to other signals such as PPG (Charlton *et al* 2015) and ECG (Lyle *et al* 2017).

## 8. Discussion and conclusions

We have described a new approach, based on attractor reconstruction, for extracting a variety of features from a blood pressure signal. The key aspects of this new approach are as follows:

• We use all of the available waveform data;
• Changes in the shape and variability of the waveform can be quantified;
• No individual points on the waveform have to be identified, which makes it more robust when dealing with noisy data;
• The only preprocessing of the data that is required is to remove any obvious outliers.

Since our approach uses all of the waveform data, this means that we can detect changes in the shape of the waveform that is not possible with any HRV method.

We note that HRV methods first extract features from the signal, most notably the beat-to-beat intervals, and then analyse these in a multitude of ways. Similarly, analysis of ECG signals typically involves identifying particular points on the signal and deriving various lengths or intervals from these points which are then averaged. An inherent problem with this approach is that it can be difficult to accurately and reliably locate the points of interest in a complex, noisy and variable signal. However, our approach does not require the identification of any individual points on the signal. We first construct an attractor in which each cycle of the data corresponds to one loop around the attractor, which is effectively an averaging process, and we then extract features from this attractor. Identifying features of the attractor, which is obtained from many cycles of the data, is much more robust than trying to identify particular points on the original signal.

We have shown that specific changes in the blood pressure waveform result in particular changes in the corresponding attractor. In table 1 we have summarised the various changes in blood pressure waveform that we have considered in this paper and the corresponding changes in the attractor for ease of reference. We have also shown how the use of artificial data can be helpful in determining the relationship between features of the signal and corresponding properties of the attractor. Of course there are many other approaches in the literature for analysing waveform data, including Fourier transforms and wavelets to mention only two. It would be interesting to compare our approach with results obtained from various other methods, but this is beyond the scope of this paper.

**Table 1.** A summary of changes in the attractor that result from particular changes in the blood pressure waveform and their physiological interpretation.

Blood pressure waveform feature	Attractor feature	Reference in paper	Physiological interpretation
Decrease in cycle length	No change in attractor but average cycle length (or heart rate) traced against time	Figure 12 (bottom)	Increase in heart rate
Increase in amplitude	Attractor size increases	Lemma 3.1 and figure 7	Increase in pulse pressure
Increased concavity of downstroke	Clockwise rotation of the attractor	Section 3.2.1	Decreased resistance and compliance of peripheral vasculature
Increased convexity of upstroke	Non-uniform density along the edges	Section 3.2.2	Increased force of cardiac contraction
Downstroke variability	Attractor consists of a thick band	Figure 17	Variability in resistance and compliance of peripheral vasculature
Upstroke variability	Variability in right hand side of attractor	Figure 16	Variability in cardiac contraction
Waveform almost periodic	Very thin sides of the attractor	Section 3	Heart rhythm almost periodic
Consistent increase/decrease in systolic and diastolic BP	No change in the attractor but change observed in the *u* variable	Section 2.3 figure 3 (top)	Overall increase/decrease in blood pressure

The next step, which we will describe elsewhere, is to use this method to identify changes in blood pressure waveform data associated with various diseases by detecting changes in the traces generated. By generating traces for multiple measures derived from the attractor, we anticipate that there will be a unique ‘signature’ in the derived traces for a variety of physiological conditions which will allow early detection of the underlying changes in the cardiovascular system control mechanisms. It is well established that for many cardiovascular diseases, earlier detection, diagnosis and more rapid clinical intervention correlates with improved patient outcome.

It has been noted for HRV methods that ‘their success in developing new clinical tools … has been so far rather limited’ (Sassi *et al* 2015). The approach that we have described above has the potential for extracting a wealth of diagnostic information from a physiological signal. However, this information will only be of benefit if it is used and applied in a clinical context. Thus, it is important moving forward to work with clinicians and health technology providers to ensure that this approach goes beyond the academic literature. Moreover, any diagnostic tool should provide outputs that are easy to interpret by clinical staff, thereby facilitating clinical decision making.

We have concentrated on analysing the reconstructed attractor after projection onto the $(v, w)$ plane, which factors out movement in the data in the vertical direction (as discussed in section 2.3). However, in any diagnostic situation, the vertical motion may also be of relevance and so further measures of baseline variation may also be useful. These could be derived from the third of our variables *u*. Our method could also be used in conjunction with the standard analysis of blood pressure signals which takes into account the vertical dimension and describes the maximum (systolic) and minimum (diastolic) pressure in each cycle, together with the reciprocal of the beat to beat interval (heart rate).

## Appendix. Proofs

Proof of lemma 2.1. From the definition of the variable *u* in (2), we have

\begin{align*} \newcommand{\e}{{\bf e}} \displaystyle \bar u&amp;=\frac{1}{3L}\int_{t^*}^{t^*+L}x(t)+y(t)+z(t)~{\rm d}t\nonumber \\ &amp;=\frac{1}{3L}\int_{t^*}^{t^*+L}x(t)+x(t-\tau)+x(t-2\tau)~{\rm d}t\nonumber \\ &amp;=\frac{1}{3L}\left(\int_{t^*}^{t^*+L}x(t)~{\rm d}t+\int_{t^*-\tau}^{t^*+L-\tau}x(t)~{\rm d}t+\int_{t^*-2\tau}^{t^*+L-2\tau}x(t)~{\rm d}t\right)\nonumber \\ &amp;=\bar x+\frac{1}{3L}\left(\int_{t^*-\tau}^{t^*}x(t)~{\rm d}t+\int_{t^*-2\tau}^{t^*}x(t)~{\rm d}t-\int_{t^*+L-\tau}^{t^*+L}x(t)~{\rm d}t-\int_{t^*+L-2\tau}^{t^*+L}x(t)~{\rm d}t\right). \nonumber \end{align*}

It is a standard result from analysis that a continuous function on a compact interval is bounded (Wade 2007), and so the maximum *M* and minimum *m* of $x(t)$ over the interval *I* exist. We then have that

\begin{align*} \newcommand{\e}{{\bf e}} \displaystyle \bar u-\bar x&amp;=\frac{1}{3L}\left(\int_{t^*-\tau}^{t^*}x(t)~{\rm d}t+\int_{t^*-2\tau}^{t^*}x(t)~{\rm d}t-\int_{t^*+L-\tau}^{t^*+L}x(t)~{\rm d}t -\int_{t^*+L-2\tau}^{t^*+L}x(t)~{\rm d}t\right)\nonumber \\ &amp;\leqslant\frac{1}{3L}(\tau M+2\tau M-\tau m-2\tau m)\nonumber \\ &amp;=(M-m)\frac{\tau}{L}. \nonumber \end{align*}

It can similarly be shown that

\begin{align*} \newcommand{\e}{{\bf e}} \displaystyle \bar u-\bar x\geqslant (m-M)\frac{\tau}{L}.\nonumber \end{align*}

Combining these two inequalities gives the stated result for $\bar u$. The results for $\bar v$ and $\bar w$ are proved in a similar way. □

Proof of theorem 2.2. It is a standard property of the Fourier transform (Pinsky 2002) that $ \newcommand{\x}{{\bf x}} {{\mathcal F}}(X(t-\tau))={\rm e}^{-2\pi {\rm i}\xi\tau}\hat X(\xi)$. Using this result and linearity of the Fourier transform gives

\begin{align*} \newcommand{\x}{{\bf x}} \newcommand{\e}{{\bf e}} \displaystyle \hat u(\xi)&amp;={{\mathcal F}}\left(\frac{1}{3}(X(t)+X(t-\tau)+X(t-2\tau))\right)\nonumber \\ &amp;=\frac{1}{3}\left({{\mathcal F}}(X(t))+{{\mathcal F}}(X(t-\tau))+{{\mathcal F}}(X(t-2\tau))\right)\nonumber \\ &amp;=\frac{1}{3}\left(\hat X(\xi)+{\rm e}^{-2\pi {\rm i}\xi\tau}\hat X(\xi)+{\rm e}^{-4\pi {\rm i}\xi\tau}\hat X(\xi)\right)\nonumber \\ &amp;=\frac{1}{3}\left(1+{\rm e}^{-2\pi {\rm i}\xi\tau}+{\rm e}^{-4\pi {\rm i}\xi\tau}\right)\hat X(\xi)\nonumber \\ &amp;=\frac{1}{3}(1+2\cos(2\pi\xi\tau)){\rm e}^{-2\pi {\rm i}\xi\tau}\hat X(\xi). \nonumber \end{align*}

The stated results for $ \newcommand{\x}{{\bf x}} \hat v(\xi)$ and $ \newcommand{\x}{{\bf x}} \hat w(\xi)$ are proved similarly. □

Proof of theorem 2.3.

(i) We define the vectors $ \newcommand{\x}{{\bf x}} \x=(x, y, z){\hspace{0pt}}^T$ and $ \newcommand{\uu}{{\bf u}} \uu=(u, v, w){\hspace{0pt}}^T$ which are related by
  \begin{align*} \newcommand{\uu}{{\bf u}} \newcommand{\x}{{\bf x}} \newcommand{\e}{{\bf e}} \displaystyle \uu=A\x\nonumber \end{align*}
  where the matrix *A* is defined by
  A.1 \begin{align*} \newcommand{\e}{{\bf e}} \displaystyle \label{Amat} A=\left(\begin{array}{@{}ccc@{}}\frac{1}{3} &amp; \frac{1}{3} &amp; \frac{1}{3} \nonumber \\[1mm] \frac{1}{\sqrt{6}} &amp; \frac{1}{\sqrt{6}} &amp; -\frac{2}{\sqrt{6}} \nonumber \\[1mm] \frac{1}{\sqrt{2}} &amp; -\frac{1}{\sqrt{2}} &amp; 0 \end{array}\right). \nonumber \end{align*}
  We note that a translation in time by one third of the period results in a permutation of the points *x*, *y* and *z*, since the signal is periodic. We now consider what effect this has on the variables *u*, *v* and *w*. Thus, we define new variables by translating backward in time by one third of the period given by
  \begin{align*} \newcommand{\uu}{{\bf u}} \newcommand{\x}{{\bf x}} \newcommand{\e}{{\bf e}} \displaystyle \tilde\x(t)=\x(t+1/3),~~\tilde\uu(t)=\uu(t+1/3)\nonumber \end{align*}
  and then clearly $ \newcommand{\x}{{\bf x}} \newcommand{\uu}{{\bf u}} \tilde\uu=A\tilde\x$ also. Since $x(t)$ has period 1 and $\tau=1/3$, we see that
  \begin{align*} \newcommand{\e}{{\bf e}} \displaystyle \tilde x(t)&amp;=x(t+1/3)=x(t-2/3)=z(t)\nonumber \\ \tilde y(t)&amp;=y(t+1/3)=x(t)\nonumber \\ \tilde z(t)&amp;=z(t+1/3)=x(t-1/3)=y(t) \nonumber \end{align*}
  and so $ \newcommand{\x}{{\bf x}} \tilde\x=C\x$ where the circulant matrix *C* is defined by
  \begin{align*} \newcommand{\e}{{\bf e}} \displaystyle C=\left(\begin{array}{@{}ccc@{}} 0 &amp; 0 &amp; 1 \nonumber \\ 1 &amp; 0 &amp; 0 \nonumber \\ 0 &amp; 1 &amp; 0 \end{array}\right)\nonumber \end{align*}
  and satisfies *C*^3^  =  *I*.
  There is a corresponding transformation in the new variables which we denote by
  A.2 \begin{align*} \newcommand{\uu}{{\bf u}} \newcommand{\e}{{\bf e}} \displaystyle \label{ueqn} \tilde\uu=R\uu \nonumber \end{align*}
  for some (invertible) matrix *R*. Combining the above results, we also see that
  \begin{align*} \newcommand{\uu}{{\bf u}} \tilde\uu=ACA^{-1}\uu \end{align*}
  Thus, we see that the transformation matrix *R* is given by
  \begin{align*} \newcommand{\e}{{\bf e}} \displaystyle R=ACA^{-1}=\left(\begin{array}{@{}ccc@{}} 1 &amp; 0 &amp; 0 \nonumber \\ 0 &amp; ~\cos\frac{2\pi}{3} &amp; \sin\frac{2\pi}{3} \nonumber \\ 0 &amp; -\sin\frac{2\pi}{3} &amp; \cos\frac{2\pi}{3} \end{array}\right).\nonumber \end{align*}
  We note that the action of the matrix *R* corresponds to a clockwise rotation in the $(v, w)$ plane by $2\pi/3$, while leaving *u* unchanged.
  Thus, moving time forwards by 1/3 corresponds to the elements of $ \newcommand{\x}{{\bf x}} \x$ cycling backwards by one position and also corresponds to a clockwise rotation in the $(v, w)$ plane by $2\pi/3$. The closed loop trajectory in the reconstructed phase space can be expressed in three parts as $ \newcommand{\x}{{\bf x}} \x(t)\cup\x(t+1/3)\cup\x(t+2/3)$ for $0\leqslant t\leqslant 1/3$. Transforming to the new coordinates and using (A.2) gives the closed loop $ \newcommand{\uu}{{\bf u}} \uu(t)\cup R\uu(t)\cup R^2\uu(t)$ for $0\leqslant t\leqslant 1/3$. In the $(v, w)$ plane, *R* acts by a clockwise rotation of $2\pi/3$ and so the closed orbit in this plane has ${\bf Z}_3$ symmetry as claimed. Clearly, the trajectory must move in the same direction as *R*, namely clockwise.
  From (A.2), it also follows from the first component that
  \begin{align*} \newcommand{\e}{{\bf e}} \displaystyle u(t+1/3)=\tilde u(t)=u(t)\nonumber \end{align*}
  and so $u(t)$ has period 1/3 as claimed.
(ii) If $\tau=1/2$, then
  \begin{align*} \newcommand{\e}{{\bf e}} \displaystyle y(t)=x(t-1/2),~~z(t)=x(t-1)=x(t)\nonumber \end{align*}
  since *x* has period 1. Thus, the transformed variables are given by
  \begin{align*} \newcommand{\e}{{\bf e}} \displaystyle v(t)&amp;=\frac{1}{\sqrt{6}}(x(t)+x(t-1/2)-2x(t-1))\nonumber \\ &amp;=\frac{1}{\sqrt{6}}(x(t-1/2)-x(t))\nonumber \\ w(t)&amp;=\frac{1}{\sqrt{2}}(x(t)-x(t-1/2)) \nonumber \end{align*}
  from which it can be seen that $v=-w/\sqrt{3}$ for all *t*.
(iii) If $\tau=2/3$, then we have
  \begin{align*} \newcommand{\e}{{\bf e}} \displaystyle y(t)&amp;=x(t-2/3)\nonumber \\ z(t)&amp;=x(t-4/3)=x(t-1/3). \nonumber \end{align*}
  Let $ \newcommand{\x}{{\bf x}} \x_{\tau}$ be the vector obtained using a time delay *τ*. Then we see that
  A.3 \begin{align*} \newcommand{\x}{{\bf x}} \newcommand{\e}{{\bf e}} \displaystyle \label{S1} \x_{2/3}=S_1\x_{1/3} \nonumber \end{align*}
  where
  \begin{align*} \newcommand{\e}{{\bf e}} \displaystyle S_1=\left(\begin{array}{@{}ccc@{}} 1 &amp; 0 &amp; 0 \nonumber \\ 0 &amp; 0 &amp; 1 \nonumber \\ 0 &amp; 1 &amp; 0 \end{array}\right)\nonumber \end{align*}
  which satisfies $S_1^2=I$. As in the proof of (i), we can then define a corresponding transformation *S*_2_ on $ \newcommand{\uu}{{\bf u}} \uu$ which satisfies $ \newcommand{\uu}{{\bf u}} \uu_{2/3}=S_2\uu_{1/3}$ by
  A.4 \begin{align*} \newcommand{\e}{{\bf e}} \displaystyle \label{S2} S_2=AS_1A^{-1} =\left(\begin{array}{@{}crc@{}} 1 &amp; 0 &amp; 0 \nonumber \\ 0 &amp; -\frac{1}{2} &amp; \frac{\sqrt{3}}{2} \nonumber \\ 0 &amp; \frac{\sqrt{3}}{2} &amp; \frac{1}{2} \end{array}\right) \nonumber \end{align*}
  where *A* is given by (A.1), which also satisfies $S_2^2=I$.
  Considering the first component, clearly this gives $u_{2/3}=u_{1/3}$ and so the transformation $\tau=1/3\to\tau=2/3$ has no effect on *u*. Since *u*_1/3_(*t*) has period 1/3, then *u*_2/3_(*t*) must also have period 1/3.
  In the $(v, w)$ plane, the transformation can be expressed as
  \begin{align*} \newcommand{\e}{{\bf e}} \displaystyle \left(\begin{array}{@{}rc@{}}-\frac{1}{2} &amp; \frac{\sqrt{3}}{2} \nonumber \\ \frac{\sqrt{3}}{2} &amp; \frac{1}{2} \end{array}\right)= \left(\begin{array}{@{}rr@{}} 1 &amp; 0 \nonumber \\ 0 &amp; -1 \end{array}\right) \left(\begin{array}{@{}rr@{}} \cos\frac{2\pi}{3} &amp; \sin\frac{2\pi}{3} \nonumber \\ -\sin\frac{2\pi}{3} &amp; \cos\frac{2\pi}{3} \end{array}\right).\nonumber \end{align*}
  Thus, the transformation $\tau=1/3\to\tau=2/3$ corresponds to a clockwise rotation by $2\pi/3$ followed by a reflection $w\to-w$ in the $(v, w)$ plane. It can be shown that this is also equivalent to a reflection in the line $w=-\sqrt{3}v$. The ${\bf Z}_3$ symmetric closed loop in the $(v, w)$ plane using $\tau=1/3$ therefore gets reflected in the *v* axis (since it is invariant under rotation by $2\pi/3$) to give a ${\bf Z}_3$ symmetric closed loop in the $(v, w)$ plane obtained using $\tau=2/3$. Consequently, the direction of the trajectory around this loop must also change, and so is anticlockwise for $\tau=2/3$.

Proof of lemma 3.1.

(i) We first consider $x(t)$ for $t\in[2/3, 1]$. In this case, using the function (14) and taking $\tau=1/3$ gives
  \begin{align*} \newcommand{\e}{{\bf e}} \displaystyle x(t)&amp;=a+\frac{3h}{2}(1-t)\nonumber \\ y(t)&amp;=a+\frac{3h}{2}\left(\frac{4}{3}-t\right)\nonumber \\ z(t)&amp;=a+3h\left(t-\frac{2}{3}\right). \nonumber \end{align*}
  If *x* and *y* lie on a straight line, it is easily verified that *w* is constant, which is the case here, and we find that *w* is given by
  \begin{align*} \newcommand{\e}{{\bf e}} \displaystyle w(t)=-\frac{h}{2\sqrt{2}}.\nonumber \end{align*}
  From the definition of *v*, we also have
  \begin{align*} \newcommand{\e}{{\bf e}} \displaystyle v(t)=\frac{\sqrt{3}h}{2\sqrt{2}}(5-6t).\nonumber \end{align*}
  We then see that $v(2/3)=\sqrt{3}h/\left(2\sqrt{2}\right)$ and $v(1)=-\sqrt{3}h/\left(2\sqrt{2}\right)$. In the $(v, w)$ plane, this corresponds to a horizontal line which is bisected by the *w* axis. As in the proof of theorem 2.3(i), we can construct the whole of the closed orbit in the $(v, w)$ plane by rotating this line by $2\pi/3$ and by $4\pi/3$, and this construction gives an equilateral triangle centred on the origin with sides of length $\sqrt{3}h/\sqrt{2}$. The trajectory follows the horizontal line given above from right to left with uniform speed ${\rm d}v/{\rm d}t=-3\sqrt{3}h/\sqrt{2}$, and so the trajectory traverses the equilateral triangle at uniform speed in a clockwise direction.
  It is easily verified from the definitions of *x*, *y* and *z* given above that $u(t)=a+h/2$ and so is constant for $t\in[2/3, 1]$. Since *u* has period 1/3 by theorem 2.3(i), then clearly $u(t)=a+h/2$ for all $t\in[0, 1]$.
(ii) If $\tau=2/3$ then we know from theorem 2.3(iii) that the orbit in the $(v, w)$ plane can be obtained by taking the orbit for $\tau=1/3$ and transforming $w\to -w$. This reflects the above equilateral triangle in the *v* axis, and the trajectory also goes in the opposite direction, i.e. anticlockwise, again at uniform speed. Also, *u* is unchanged and so we again have $u(t)=a+h/2$.

Proof of lemma 3.2. Since $\tau=1/3$, for $t\in[2/3, 1]$, *x* and *y* are both traversing the second component of *x* while *z* moves along the first component. It is a matter of calculation to show that the variables *v* and *w* on this time interval are given by

\begin{align*} \newcommand{\e}{{\bf e}} \displaystyle \begin{array}{@{}ll@{}} v(t)=-\frac{\beta h}{2\sqrt{2}}+\frac{\sqrt{3}h}{2\sqrt{2}}(5-6t), &amp; \frac{2}{3}\leqslant t\leqslant 1 \nonumber \\[3mm] w(t)=-\frac{h}{2\sqrt{2}}-\frac{\sqrt{3}\beta h}{2\sqrt{2}}(5-6t), &amp; \frac{2}{3}\leqslant t\leqslant 1. \end{array}\nonumber \end{align*}

Eliminating *t* from the equations for *v* and *w* gives the stated straight line in the $(v, w)$ plane. Since *v* and *w* are linear functions of *t*, the motion along this edge occurs at uniform speed. The length of the side of the triangle is then easily calculated. The range of values of *β* for which the first quadratic function in *x* is monotonic can be found by assuming that the turning point of the quadratic occurs at either *t*  =  0 or *t*  =  1/3. A similar approach can be used for the second quadratic function in *x*, where it is assumed that the turning point occurs at either *t*  =  1/3 or *t*  =  1. When $\beta=0$, the two functions are linear and hence have no turning point, and so the range of values between the two calculated values is the range for which there are no turning points in the interval of interest, resulting in a monotonic function. □

Proof of lemma 3.3. Using the definitions of *v* and *w*, we find in this case that *v* is a quadratic function and *w* a linear function of *t* for $2/3\leqslant t\leqslant 1$. Solving for *t* in terms of *w* and substituting into *v* gives *v* as a quadratic function of *w* with the stated quadratic coefficient *A*. If *x*_1_ is convex or linear and *x*_2_ is concave, then $a_1\leqslant 0$ and *a*_2_  >  0 and so *A*  >  0 as claimed. It can also be shown that $w(1)-w(2/3)=\sqrt{2}a_2/9$ and $v(1)-v(2/3)=-\sqrt{3}h/\sqrt{2}$. If *x*_2_ is concave, then *a*_2_  >  0 and so $w(1)&gt;w(2/3)$, so that the straight line joining these corner points has a negative slope, which again corresponds to a clockwise rotation of the corner points of the attractor. The slope of the straight line between the two end points of this quadratic curve has slope $(w(1)-w(2/3))/(v(1)-v(2/3))=-2a_2/(9\sqrt{3}h)$ as claimed. Since $\tau=1/3$, by theorem 2.3(i) the closed trajectory in the $(v, w)$ plane has ${\bf Z}_3$ symmetry and so the remaining two edges of the closed orbit can be obtained by rotations by $2\pi/3$ and $4\pi/3$ about the origin. □
